# Inflammatory phenotypes in severe pneumonia: clinical evidence to mouse models for precision therapeutics

**DOI:** 10.1093/ajrccm/aamag402

**Published:** 2026-07-31

**Authors:** Hiroki Taenaka, Bruno Evrard, Mazharul Maishan, Ruriko Watanabe, Narges Alipanah-Lechner, Sidney A Carrillo, Aartik Sarma, Natasha Spottiswoode, Kathryn M Sullivan, Melanie F Weingart, Hanjing Zhuo, Angelika Ringor, Carolyn Leroux, Olivia Chao, Chelsea Lin, Emma Schmiege, Taarini Hariharan, Liam Magee, Andrew Willmore, Suzanna Chak, Deanna Lee, Kim Bardillon, Carolyn Hendrickson, Jeffrey E Gotts, Charles R Langelier, Carolyn S Calfee, Michael A Matthay

**Affiliations:** Cardiovascular Research Institute, University of California, San Francisco, CA, United States; Department of Anesthesiology and Intensive Care Medicine, Osaka University Graduate School of Medicine, Suita, Japan; Inserm CIC 1435, Dupuytren Teaching Hospital, Limoges, France; Department of Medicine, University of California, San Francisco, CA, United States; Department of Anesthesia, Cardiovascular Research Institute, University of California, San Francisco, CA, United States; Cardiovascular Research Institute, University of California, San Francisco, CA, United States; Cardiovascular Research Institute, University of California, San Francisco, CA, United States; Department of Medicine, University of California, San Francisco, CA, United States; Department of Medicine, University of California, San Francisco, CA, United States; Department of Medicine, University of California, San Francisco, CA, United States; Department of Medicine, University of California, San Francisco, CA, United States; Department of Medicine, University of California, San Francisco, CA, United States; Department of Medicine, University of California, San Francisco, CA, United States; Cardiovascular Research Institute, University of California, San Francisco, CA, United States; Department of Medicine, University of California, San Francisco, CA, United States; Department of Anesthesia, Cardiovascular Research Institute, University of California, San Francisco, CA, United States; Department of Medicine, University of California, San Francisco, CA, United States; Department of Anesthesia, Cardiovascular Research Institute, University of California, San Francisco, CA, United States; Department of Medicine, University of California, San Francisco, CA, United States; Department of Anesthesia, Cardiovascular Research Institute, University of California, San Francisco, CA, United States; Department of Medicine, University of California, San Francisco, CA, United States; Department of Anesthesia, Cardiovascular Research Institute, University of California, San Francisco, CA, United States; Department of Medicine, University of California, San Francisco, CA, United States; Department of Anesthesia, Cardiovascular Research Institute, University of California, San Francisco, CA, United States; Department of Medicine, University of California, San Francisco, CA, United States; Department of Anesthesia, Cardiovascular Research Institute, University of California, San Francisco, CA, United States; Department of Medicine, University of California, San Francisco, CA, United States; Department of Anesthesia, Cardiovascular Research Institute, University of California, San Francisco, CA, United States; Department of Medicine, University of California, San Francisco, CA, United States; Department of Anesthesia, Cardiovascular Research Institute, University of California, San Francisco, CA, United States; Department of Medicine, University of California, San Francisco, CA, United States; Department of Medicine, University of California, San Francisco, CA, United States; Department of Medicine, University of California, San Francisco, CA, United States; Department of Medicine, University of California, San Francisco, CA, United States; Department of Medicine, University of California, San Francisco, CA, United States; Department of Medicine, University of California, San Francisco, CA, United States; Cardiovascular Research Institute, University of California, San Francisco, CA, United States; Department of Medicine, University of California, San Francisco, CA, United States; Department of Anesthesia, Cardiovascular Research Institute, University of California, San Francisco, CA, United States; Cardiovascular Research Institute, University of California, San Francisco, CA, United States; Department of Medicine, University of California, San Francisco, CA, United States; Department of Anesthesia, Cardiovascular Research Institute, University of California, San Francisco, CA, United States

**Keywords:** Pneumonia, acute respiratory distress syndrome, sepsis

## Abstract

**Rationale:**

Hyperinflammatory and hypoinflammatory phenotypes previously identified in sepsis and acute respiratory distress syndrome may enable precision therapies, but their clinical relevance and translational modeling in severe pneumonia remain incompletely characterized.

**Objectives:**

To examine biomarker-defined hyperinflammatory and hypoinflammatory phenotypes in critically ill patients with pneumonia (pulmonary sepsis), test whether the biomarkers that define these phenotypes identify subgroups and outcomes in a mouse model of bacterial pneumonia, and determine whether the mouse phenotypes respond differently to therapeutic interventions.

**Methods:**

We performed latent class analysis (LCA) in a cohort of 548 ICU patients with pulmonary sepsis to identify inflammatory phenotypes and test the association of these phenotypes with clinical outcomes using validated classifier models. We developed a mouse model of pneumococcal pneumonia that recapitulated key aspects of these phenotypes and tested responses to dexamethasone and IL-6 receptor blockade.

**Measurements and Main Results:**

Two phenotypes were identified in patients with pulmonary sepsis: a hyperinflammatory phenotype with more lung injury and increased mortality, and a hypoinflammatory phenotype with more favorable outcomes. Despite uniform pathogen exposure and baseline conditions, LCA identified 2 phenotypes in the mice with divergent trajectories (lung injury and mortality). Therapeutic benefit from anti-inflammatory interventions was observed exclusively in the more inflamed mice.

**Conclusions:**

By integrating clinical data from human studies and experimental findings in a clinically relevant mouse model, this study provides a translational framework for developing phenotype-targeted therapies for critical illness, with the goal of improving patient outcomes.

## Introduction

Pneumonia remains a leading cause of mortality worldwide^1^ and is the most common infectious cause of hospitalization and death among adults in the United States.^2^ Driven by infection and inflammation, it can progress to severe complications including sepsis, acute respiratory distress syndrome (ARDS), and death.^3^^,^^4^ Despite decades of research, treatment of critically ill patients with pneumonia still relies primarily on antibiotics, and host-directed therapies have largely failed to materialize.^5^

Repeated trial failures have spurred interest in personalized medicine for pneumonia.^6^ Prognostic and predictive enrichment strategies are increasingly recognized as essential for rational therapy development, offering pathways to better-targeted treatments.^7^ Enrichment based on biological phenotyping has been studied in ARDS and sepsis.^8-10^ For example, the “hyperinflammatory” and “hypoinflammatory” phenotypes identified by our group and others^11^^,^^12^ using latent class analysis (LCA) respond differently to therapies including mechanical ventilation, fluid management, simvastatin, activated protein C, and corticosteroids.^10^^,^^13-15^ However, such strategies remain understudied in cohorts defined by severe pneumonia.

Preclinical studies have likewise rarely applied personalized medicine strategies in animal models of ARDS or pneumonia. Identifying the biological pathways underlying human phenotypes is essential for clinical relevance, yet observational studies in patients alone cannot elucidate the pathways driving phenotype-specific outcomes or test potential therapies. Addressing these gaps requires in vivo approaches that prioritize therapies tailored to each phenotype’s underlying mechanism.^7^

Building on previous bedside-to-bench research that translated pediatric sepsis endotypes into clinically relevant mouse models,^16^ this research had 3 objectives: (1) to examine the distribution and clinical relevance of biomarker-defined hyperinflammatory and hypoinflammatory phenotypes previously identified in ARDS and sepsis within a critically ill cohort with pneumonia and pulmonary sepsis; (2) to test whether the biomarkers defining these phenotypes identify subgroups and/or predict acute lung injury outcomes in our well-established mouse model of severe bacterial pneumonia^17^; and (3) to determine whether the resulting mouse phenotypes respond differently to clinically relevant therapeutic interventions.

## Methods

### Study design: patient cohort

The Early Assessment of Renal and Lung Injury (EARLI) cohort is a prospective observational study enrolling patients admitted from the emergency department to the ICU at 2 San Francisco, CA, hospitals: a tertiary referral center and an urban safety-net hospital. Patients were enrolled between 2009 and December 2024. The University of California San Francisco (UCSF) Institutional Review Board approved the study (protocol #10-02852), and patients or their surrogates provided informed consent, with exceptions as previously described.^17^ The day of ICU admission was study day 1. Blood samples for biomarker measurements were obtained within 24 hours of ICU admission. From the broader EARLI cohort, we selected patients presenting with pneumonia upon ICU admission and a chest x-ray indicative of community-acquired pneumonia, confirmed independently by at least 2 physicians. Sepsis was determined using Sepsis-3 criteria by independent physician review of electronic medical records. Methods for the parsimonious classifiers and patient microbiology assessments are found in the online supplementary material.

### Biomarker measurements and latent class analysis in patients

LCA was applied to clinical and biological variables to identify inflammatory phenotypes in patients. Variables (Table S1; see online supplementary material) were selected based on prior studies to enable comparison with phenotypes previously described in ARDS and sepsis. Biomarkers were measured under standardized protocols. Models with 1 to 4 classes were evaluated, and the optimal model was selected by fit indices, including the Bayesian information criterion (BIC), entropy, and the Vuong-Lo-Mendell-Rubin likelihood ratio test. See online supplementary material for additional details.

### Mouse model of pneumonia

All animal experiments were approved by the UCSF Institutional Animal Care and Use Committee (protocol no. AN189182). Female wild-type C57BL/6 mice age 9 to 12 weeks (National Cancer Institute, Frederick, MD) were inoculated intranasally with *Streptococcus pneumoniae* serotype 19F at a dose of 2 × 10^8^ colony-forming units (CFU). To enhance clinical relevance, mice received intraperitoneal ceftriaxone (150 mg/kg) at 18 and 30 hours postinoculation, corresponding to the onset of hypoxemia and intended to model delayed treatment initiation in clinical practice.^18^ For therapeutic studies, mice were randomized to dexamethasone or an IL-6 receptor antibody, each combined with ceftriaxone. Control groups received ceftriaxone plus vehicle. We used 110 mice for the biomarker-prognosis analysis. Separately, we used 60 mice for the dexamethasone study (30 vs 30), and 60 mice for the IL-6 receptor antibody study (30 vs 30). A small number were excluded for inadequate biomarker sampling (3 in the biomarker-prognosis analysis, 4 in the dexamethasone study).

Body temperature, body weight, and oxygen saturation (SpO_2_) were measured before inoculation at baseline (H0) and at 18 and 36 hours postinoculation. At 36 hours, mice were sacrificed, and lung injury was assessed using bronchoalveolar lavage (BAL) for protein concentration and bacterial titers. Mice that died before 36 hours or were euthanized earlier per prespecified humane endpoints did not undergo sample collection. See online supplementary material for additional details.

### Biomarker sampling and latent class analysis in mice

Plasma for LCA was collected at 18 hours postinfection, before any therapeutic intervention, corresponding to the onset of hypoxemia and an early disease stage. All biomarkers were measured retrospectively using standardized procedures, allowing subsequent phenotype classification while investigators remained blinded to phenotype during the experiments. Analyses used the pooled data set across all experimental batches to avoid bias from small batch-specific sample sizes. LCA was performed using inflammatory biomarkers (IL-6, keratinocyte-derived chemokine [KC], sTNFR-1, and matrix metalloproteinase-8 [MMP-8]) and physiological variables. Models with 1 to 4 classes were evaluated, and the optimal model was selected by the Bayesian information criterion (BIC), entropy, and the Vuong-Lo-Mendell-Rubin (VLMR) likelihood ratio test. Each mouse was assigned to a class by highest posterior probability.

### Statistical analyses

For patients and mice, continuous variables were summarized as medians and interquartile ranges. Comparisons between classes and between treatment and control groups used Mann-Whitney U or chi-square tests, with false discovery rate correction for multiple testing where appropriate. Correlations were evaluated using Spearman’s rank correlation. All tests were 2-sided, with *P* value < .05 considered significant. Analyses were conducted in R (version 4.3.2) and, for LCA, Mplus (Muthen and Muthen, version 1.8.11); see online supplementary material for additional details.

## Results

### Critically ill patients with pulmonary sepsis

The 548 pulmonary sepsis patients included from EARLI (Figure 1; Figure S1, see online supplementary material for Figure S1) had a median age of 65 years; 64% were male, and 527 (96%) met Sepsis-3 criteria (Table 1; Table S2, see online supplementary material). At ICU admission, 265 patients (48%) were receiving mechanical ventilation, and 246 (45%) required vasopressors; 239 (44%) developed ARDS within 5 days by Berlin criteria. Overall ICU mortality was 20%.

**Figure 1:**
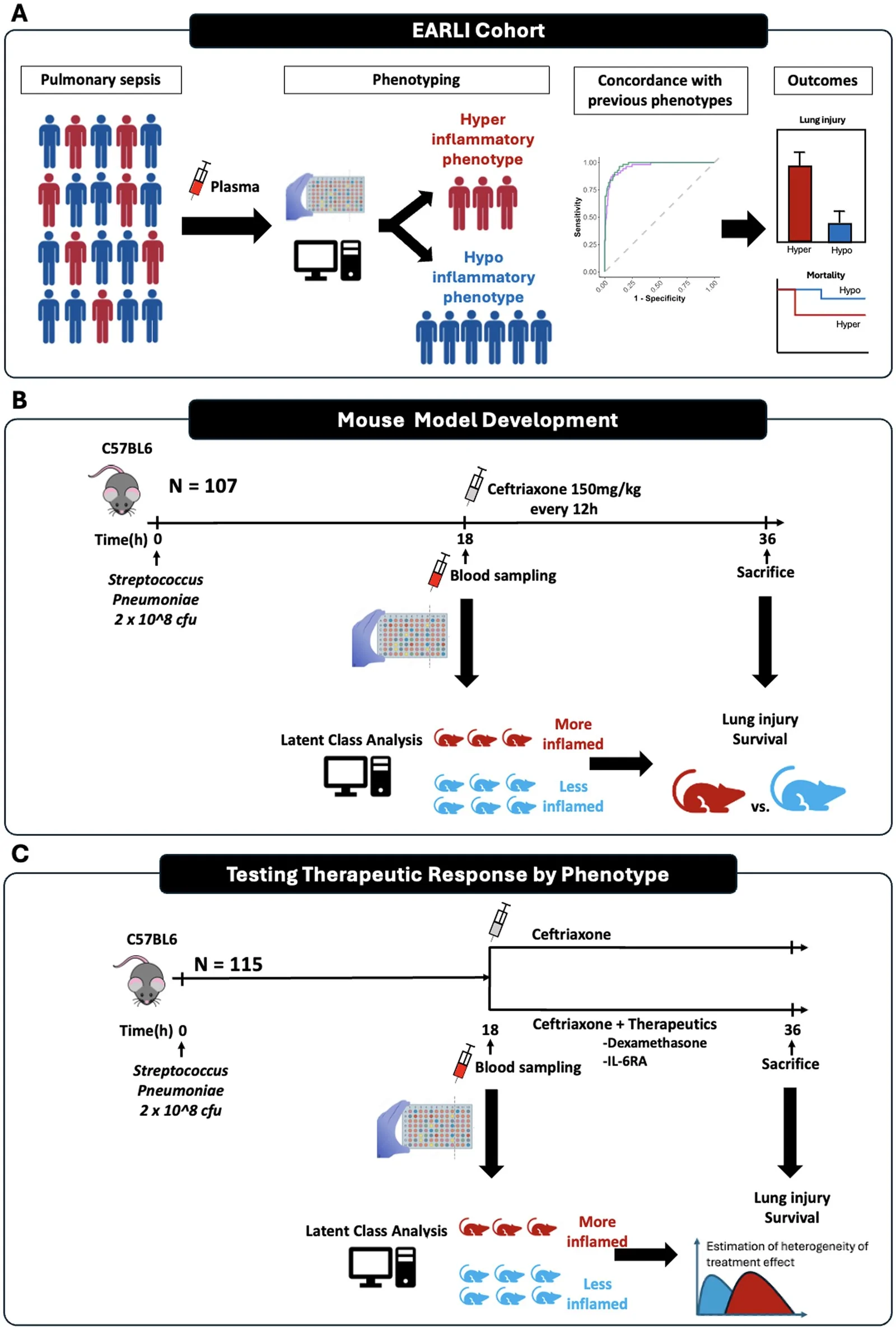
Overview of the protocol and approach used. (A) We first conducted latent class analysis (LCA) using clinical and biomarker data from a prospective cohort of critically ill patients with pneumonia to confirm that inflammatory phenotypes could be identified in pulmonary sepsis and were associated with different outcomes. (B) In the mouse model development, 107 female mice, across 4 different batches, were infected with *Streptococcus pneumoniae* and treated with antibiotics at 18 and 30 hours postinoculation. Mice were sacrificed at 36 hours. Inflammatory phenotypes (represented by different colors) were retrospectively identified using biomarkers measured at 18 hours analyzed with LCA. The characteristics of the mice were then analyzed based on LCA classification. (C) In the testing of the therapeutic agents model, experiments were conducted using dexamethasone or an IL-6 receptor antagonist in combination with antibiotics in 55 mice (dexamethasone) and 60 mice (IL-6 receptor antagonist) respectively. A new LCA was performed to identify inflammatory phenotypes, including all mice (from B + C), and heterogeneity of treatment effect was evaluated independently for each treatment.

**Table 1:** Characteristics of patients with severe pneumonia population stratified by phenotype.

Characteristic	Overall *n* = 548^a^	Hypoinflammatory *n* = 451^a^	Hyperinflammatory *n* = 97^a^	*P* value^b^	Adjusted *P* value^c^
**Men**	349 (64%)	285 (63%)	64 (66%)	.6	.7
**Age**	65 (54, 75)	66 (55, 76)	60 (49, 71)	.002	.003
**SOFA**	8 (5, 12)	7 (4, 10)	13 (11, 16)	<.001	<.001
**Ethnicity**				.6	.6
*** Caucasian***	257 (47%)	215 (48%)	42 (43%)		
*** African American***	80 (15%)	61 (14%)	19 (20%)		
*** Asian***	140 (26%)	115 (25%)	25 (26%)		
*** Other***	71 (13%)	60 (13%)	11 (11%)		
**APACHE III**	86 (63, 118)	81 (59, 108)	137 (105, 163)	<.001	<.001
**Mechanical ventilation at enrollment**	265 (48%)	197 (44%)	68 (70%)	<.001	<.001
**Vasopressors at enrollment**	246 (45%)	167 (37%)	79 (81%)	<.001	<.001
**Sepsis-3 criteria met**	527 (96%)	430 (95%)	97 (100%)	.035	.042
**ARDS at enrollment (Berlin definition)**	239 (44%)	168 (37%)	71 (73%)	<.001	<.001
**PaO_2_/FiO_2_ (mmHg)**	173 (91, 258)	192 (110, 272)	111 (77, 180)	<.001	<.001
**Biomarkers**					
**IL-6 *(pg/mL)***	83 (19, 487)	57 (15, 171)	3450 (730, 8880)	<.001	<.001
**IL-8 *(pg/mL)***	16 (7, 62)	12 (6, 26)	635 (185, 2336)	<.001	<.001
**Ang-2 *(pg/mL)***	5674 (3061, 11 285)	4754 (2591, 9267)	15 154 (7604, 22 187)	<.001	<.001
**RAGE *(pg/mL)***	2834 (1598, 5182)	2553 (1521, 4878)	4816 (2889, 8686)	<.001	<.001
**ICAM-1 *(pg/mL)***	635 526 (384 935, 981 236)	588 882 (369 788, 873 504)	1 013 021 (501 886, 1 550 439)	<.001	<.001
**TNFR-1 *(pg/mL)***	3309 (1738, 7125)	2804 (1525, 5094)	8379 (4442, 12 830)	<.001	<.001
**PAI-1 *(pg/mL)***	5 (3, 14)	4 (2, 9)	34 (17, 78)	<.001	<.001
**Protein C (% control)**	73 (44, 111)	81 (52, 116)	38 (17, 71)	<.001	<.001
**ICU mortality**	109 (20%)	59 (13%)	50 (52%)	<.001	<.001

a *n* (%); median (Q1, Q3).

b Pearson’s chi-square test; Mann-Whitney U test.

c False discovery rate correction for multiple testing.

Abbreviations: APACHE III, Acute Physiology and Chronic Health Evaluation III; Ang-2, angiopoietin-2; ARDS, acute respiratory distress syndrome; ICAM-1, intercellular adhesion molecule-1; RAGE, receptor for advanced glycation end products; SOFA, Sequential Organ Failure Assessment.

A 2-class latent class model provided the best fit (Table S3, see online supplementary material); 451 patients (82%) were classified as hypoinflammatory and 97 (18%) as hyperinflammatory (Table 1; Table S2, see online supplementary material).

Seven plasma biomarkers were compared between groups. The hyperinflammatory phenotype had higher proinflammatory biomarkers (IL-6, IL-8, sTNFR-1; Figure 2A, 2B, Table 1), lower serum bicarbonate, and more frequent vasopressor use than the hypoinflammatory phenotype. These characteristics are consistent with the phenotypes previously identified in ARDS and sepsis.^8^^,^^10^^,^^14^^,^^19^ As in those studies, hyperinflammatory patients had significantly worse outcomes, including fewer ventilator-free days and higher ICU mortality (52% vs 13%; *P* < .001) (Table 1, Figure 2C). In a sensitivity analysis excluding patients meeting ARDS criteria, the latent class structure and associated characteristics remained consistent with the overall cohort (Table S4, Figure S2; see online supplementary material), with preserved discrimination (entropy = 0.91), supporting the robustness of these phenotypes beyond ARDS.

**Figure 2:**
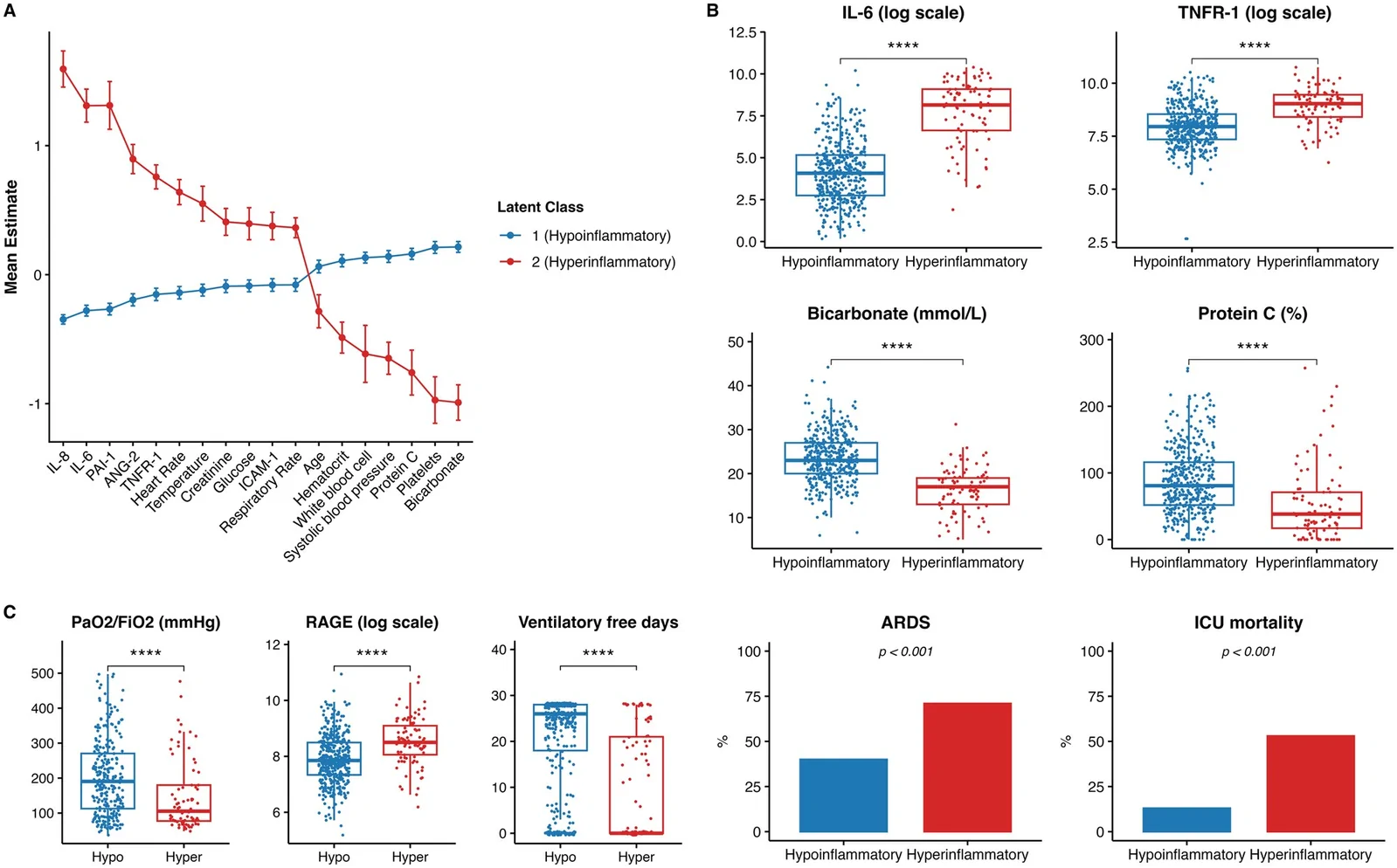
Identification and clinical characterization of inflammatory phenotypes in critically ill patients with pulmonary sepsis. (A) Standardized mean values of continuous variables stratified by class after latent class analysis (LCA), with corresponding standard errors. (B) The box plots compared the main biomarkers used for the LCA stratified by identified class. Given the profile, we identified a hypoinflammatory phenotype (class 1, in blue) and a hyperinflammatory phenotype (class 2, in red). (C) The box plots compare the outcome and severity, stratified by phenotype. Individual data points are displayed as jittered dots, and statistical comparisons between groups are indicated. Continuous variables were compared using Mann-Whitney U test, and categorical variables were compared using the chi-square test. **** *P* value < .001. Abbreviations: ARDS, acute respiratory distress syndrome; ANG-2, angiopoietin-2; ICAM-1, intercellular adhesion molecule 1; RAGE, receptor for advanced glycation end products.

Previously validated parsimonious classifier models showed high concordance with the LCA-derived phenotypes (Table S5, see online supplementary material). Clinical microbiology tests identified common pneumonia pathogens, with *S. pneumoniae* as the most common bacterial pathogen and influenza the most common viral pathogen (Table S6, see online supplementary material). Hyperinflammatory patients were more likely to have a causative pathogen identified (Table S6, see online supplementary material).

### Latent class analysis identifies 2 phenotypes in mice with pneumonia

After confirming that inflammatory phenotypes were observable in patients with pneumonia, we next investigated whether similar heterogeneity existed in a mouse model of pneumococcal pneumonia. Plasma inflammatory biomarkers measured at 18 hours postinfection (IL-6, KC/IL-8, MMP8, and sTNFr1) reflected variable severity of the inflammatory response in mice and were associated with subsequent lung injury severity (Figure 3A; see online supplementary material for supplemental results). Given this variance, we asked whether LCA would identify biological phenotypes in mice with pneumococcal pneumonia. First, we studied mice treated only with antibiotics (*n* = 107) to avoid confounding by pharmacotherapy. The mouse LCA used 18-hour data: specifically, the 4 inflammatory biomarkers analogous to the human LCA, body weight, and presence of bacteremia. This time point corresponded to the onset of hypoxemia and the first antibiotic dose. As in the human data, the 2-class model provided the best fit (Table S7, see online supplementary material; VLMR test, *P* = .0286) and identified 2 classes (phenotypes) distinguished primarily by the severity of the inflammatory response (Figure 3B, 3C). Addition of Ang-2 and ICAM-1 had minimal impact on the model or class assignments (Tables S8 and S9, see online supplementary material).

**Figure 3:**
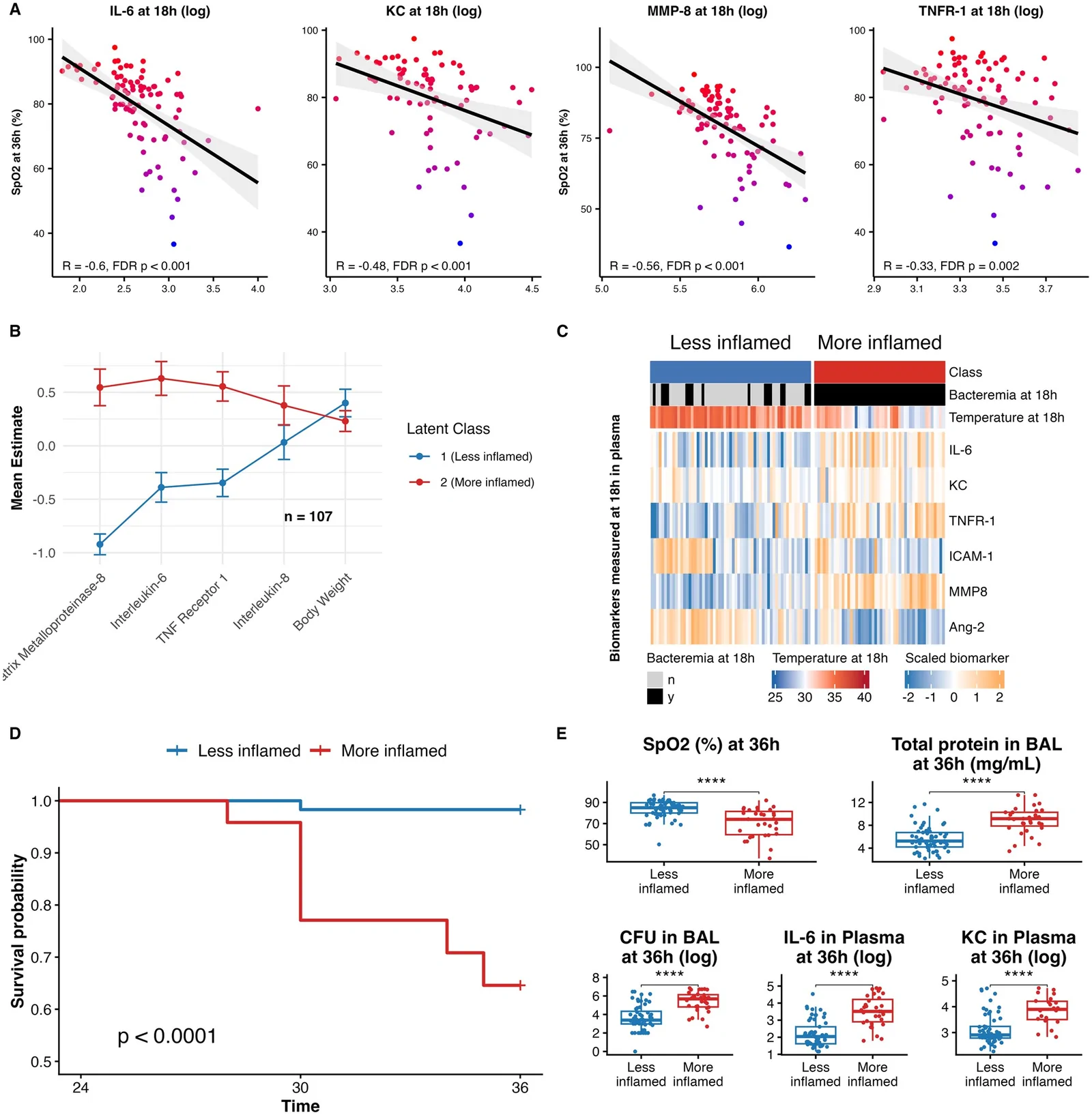
Identification of inflammatory phenotypes in mice with pneumococcal pneumonia. (A) Correlation between plasma biomarkers at 18 hours and arterial oxygen saturation. The scatter plots include a regression line (black) with a 95% confidence interval (gray shading). Colors indicate values of *y*-axis variables, ranging from red (higher) to blue (lower). All biomarker values are log-transformed. Spearman correlation coefficients were used to assess associations, and *P* values were adjusted using false discovering rate correction for multiple testing. (B) Standardized mean values of continuous variables at 18 hours stratified by class after latent class analysis (LCA) in the development phase model, with corresponding standard errors. (C) The heat map shows the z-scored, log-transformed levels of biomarkers measured in mice from the development model at 18 hours after infection, grouped by LCA classes. Each column represents 1 mouse; each row corresponds to 1 biomarker. Top annotations indicate the presence of bacteremia at 18 hours postinfection and body temperature. Color intensity within the heat map reflects standardized expression levels (z-scores): blue indicates below-average, red above-average expression. (D) Kaplan-Meier survival curves illustrating the probability of survival over time for each class. (E) Box plots comparing oxygen saturation (SpO_2_) levels, total protein levels in BAL, bacterial load in BAL, IL-6 and KC in plasma, all measured 36 hours postinfection, between less inflamed class (blue) and more inflamed class (red). Variables were compared using Mann-Whitney U test. **** *P* value < .001. Abbreviations: ANG-2, angiopoietin-2; CFU, colony-forming unit; ICAM-1, intercellular adhesion molecule 1; KC, keratinocyte-derived chemokine; MMP-8, matrix metalloproteinase-8; RAGE, receptor for advanced glycation end products.

At baseline (H0), the phenotypes later classified as less or more inflamed differed only in body weight (Table S10, see online supplementary material). At 18 hours, SpO_2_ did not differ, but body temperature was lower in the more inflamed phenotype; bacteremia was universal in the more inflamed phenotype (*n* = 48; 100%) but not in the less inflamed phenotype (*n* = 16; 27%; *P* < .001). At 36 hours, mice in the more inflamed phenotype had lower survival (Figure 3D); survivors were more hypoxemic (Figure 3E) and had more severe lung injury, reflected by higher BAL total protein (Figure 3E). Also at 36 hours, plasma and BAL IL-6 and KC, and BAL bacterial load, were significantly higher in the more inflamed phenotype (Table S10, see online supplementary material). These results indicate strong concordance between the more inflamed mouse phenotype and the hyperinflammatory phenotype in critically ill patients with pulmonary sepsis (Table 1; Table S10, see online supplementary material). Interestingly, plasma sICAM-1 and Ang-2 were lower in the less inflamed mice, in contrast to humans, in which both markers tended to be higher in the hyperinflammatory phenotype.^8^^,^^20^

Having defined baseline phenotypes without therapeutic intervention, we extended the same LCA framework to the larger mouse cohort (*n* = 222) that included the pharmacologic interventions explained in the next section. This extended LCA again identified a 2-class solution as the best fit, with features similar to the development phase (Table S11, Figure S3; see online supplementary material); adding Ang-2 and ICAM-1 had minimal impact on the model or class assignments (Tables S12 and S13, see online supplementary material).

### Differential effect of therapeutics on outcomes stratified by phenotype in the mice

We next tested whether the response to 2 anti-inflammatory therapies, dexamethasone and IL-6 receptor antibody (IL6RA), differed by phenotype in mice.

#### Dexamethasone

Of 55 mice studied, 28 (51%) were randomized to control and 27 (49%) to dexamethasone (Table S14, see online supplementary material). Plasma IL-6, KC (IL-8), and TNFR-1 decreased between 18 and 36 hours postinfection in both groups (Figure S4, see online supplementary material). Dexamethasone reduced plasma and BAL IL-6 and TNFR-1 (Table S15, Figure S4; see online supplementary material).

Across all mice, dexamethasone significantly improved SpO_2_ and BAL total protein at 36 hours (Table S15, see online supplementary material; Figure 4A, 4B). When mice were stratified by phenotype, these improvements were only observed in the more inflamed phenotype (Figure 4A, 4B). The interaction between phenotype-assignment probability (as a continuous variable) and outcome was significant for SpO_2_ (*P* = .0035) (Figure 4A), but not BAL total protein (*P* = .235) (Figure 4B).

**Figure 4:**
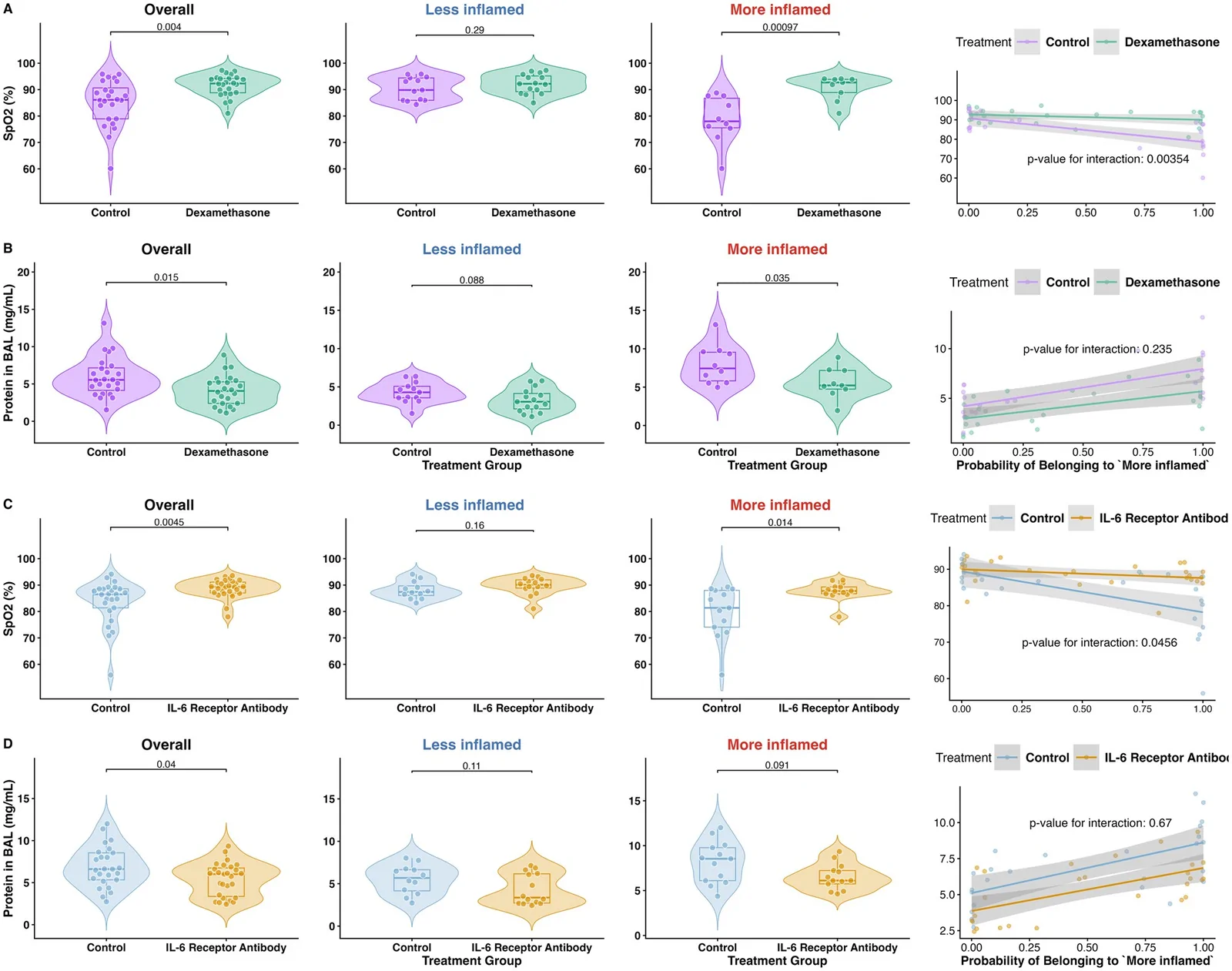
Effect of dexamethasone and IL-6 receptor antibody on SpO_2_ and BAL protein levels. Box plots compare SpO_2_ levels (A and C) and BAL protein levels (B and D) between the control and dexamethasone-treated or IL-6 receptor antibody groups, stratified by inflammatory classes (less inflamed vs more inflamed class). Individual data points are displayed as jittered dots. Variables were compared using Mann-Whitney U test. On the right, scatter plot shows the relationship between the probability of belonging to more inflamed class and SpO_2_ or BAL proteins level at 36 hours, with a fitted regression line. The *P* value for the interaction between treatment and class is annotated on the figure. Abbreviations: IL-6RA, IL-6 receptor antibody; SpO_2_, oxygen saturation.

#### IL-6 receptor antibody

Of 60 mice, 30 (50%) were randomized to control and 30 (50%) to IL6RA (Table S16, see online supplementary material). As expected,^21^ plasma IL-6 significantly increased after IL6RA administration in both phenotypes (Figure S5, Table S17; see online supplementary material).

As with dexamethasone, IL6RA improved SpO_2_ and BAL total protein at 36 hours across all groups (Figure 4C, 4D); when stratified by phenotype, benefit was observed only in the more inflamed phenotype (Figure 4C, 4D). The interaction between phenotype and outcome was again significant for SpO_2_ (*P* = .0456) but not BAL total protein (*P* = .67) (Figure 4C, 4D). These findings suggest that anti-inflammatory therapies such as steroids and IL6RA may be more effective in the more inflamed phenotype. Adjusting for baseline body weight did not meaningfully alter the results of either analysis (Tables S18 and S19; see online supplementary material).

### Heterogeneity across batches

To interrogate sources of variation in the inflammatory response within mice, we examined the heterogeneity across batches (Figure S6, see online supplementary material). Severity varied widely across all batches, although some exhibited more severe patterns, as characterized by lower SpO_2_, higher BAL total protein, and elevated plasma IL-6 at 36 hours. These batches also had a higher proportion of more inflamed mice and increased mortality. Importantly, both phenotypes appeared within all batches, indicating that phenotype assignment was not batch-restricted (Figure S7, see online supplementary material). Baseline body temperature and body weight showed no distinct batch patterns. To test whether variation related to bacterial load despite a uniform inoculum, we infected 10 mice with 2 x 10^8^  *S. pneumoniae* using the standard protocol and sacrificed them at 1 hour to measure BAL bacterial load. Load did not vary significantly across mice (Figure S8, see online supplementary material), indicating that inoculum differences were unlikely to explain the observed variance in severity.

In sensitivity analyses incorporating batch as a random effect in a mixed-effects model, the association between phenotype and treatment response was unchanged after adjustment for batch (Tables S20 and S21, see online supplementary material), indicating robustness to batch effects.

Although female mice were used for most studies, experiments using the same protocol in 28 male mice showed the same heterogeneity of response to bacterial pneumonia, confirming that results were not sex-specific (Figure S9, see online supplementary material).

## Discussion

This bedside-to-bench study has 2 major findings. First, the hyperinflammatory and hypoinflammatory phenotypes previously identified in sepsis and ARDS are relevant in critically ill patients with pneumonia and pulmonary sepsis. Notably, the hyperinflammatory phenotype was associated with higher mortality, elevated plasma inflammatory biomarkers, and different pathogen patterns, highly concordant with observations of this phenotype in ARDS and sepsis. Second, the in vivo mouse pneumonia model likewise identified 2 inflammatory phenotypes, “more inflamed” and “less inflamed,” with divergent lung injury severity and differential responsiveness to corticosteroids and IL-6 receptor antagonists. Together, these findings suggest that a gradient of inflammatory host responses may be conserved across syndromic definitions of critical illness and across species, even with a uniform pathogen inoculum. Although directly equating mouse and human phenotypes warrants caution, the consistent association between a more robust inflammatory response, greater severity, and differential treatment responsiveness supports the hypothesis that inflammatory phenotyping represents a clinically relevant and potentially treatable trait.^22^

### Inflammatory phenotypes in critically ill patients with pulmonary sepsis

LCA identified 2 distinct inflammatory phenotypes in critically ill patients with pneumonia and pulmonary sepsis, consistent with previous studies in sepsis and ARDS and underscoring the interconnected nature of these conditions. Pneumonia is a primary cause of ARDS, a significant trigger for sepsis, and a leading reason for ICU admission.^23^^,^^24^ Identifying these phenotypes in our cohort does not represent discovery of new clinical subgroups; rather, the human analysis establishes a clinically relevant reference framework for the experimental mouse model. Whereas prior studies described these phenotypes in broader sepsis populations of mixed etiology, our study focuses on a cohort restricted to pneumonia and pulmonary sepsis, evaluating the phenotypes within a more homogeneous clinical entity and allowing clearer interpretation of host-response heterogeneity.

The proportion of the hyperinflammatory phenotype in our human cohort was lower than in prior ARDS and sepsis studies, suggesting that phenotype distribution varies by underlying etiology and consistent with reports that respiratory infections are a major driver of hypoinflammatory ARDS and sepsis.^10^^,^^25^ One possible explanation is compartmentalization of inflammation within the lungs in the hypoinflammatory phenotype. Although we did not measure alveolar protein directly, prior studies support this hypothesis: Bendib et al. reported elevated IL-8 in the alveolar space of patients with pneumonia-related ARDS while systemic levels remained low,^26^ a gradient that likely shifts depending on pneumonia severity and pathogen. Consistent with localized infection, fewer than 10% of hypoinflammatory patients had positive blood cultures, whereas hyperinflammatory patients had higher rates, indicating systemic inflammation likely driven by circulating pathogens. Pathogen patterns also differed significantly: hyperinflammatory patients were more frequently infected with *Streptococcus pneumoniae* and *Enterobacter*, pathogens that differ markedly in genetic and biologic properties yet appear to elicit distinctly potent inflammatory responses.^27^

An important question is whether human hyperinflammatory and hypoinflammatory phenotypes represent distinct biological subgroups or different stages along a common disease trajectory. In pneumonia, infection typically begins in the lung and, in some patients, progresses to a systemic inflammatory state, making it plausible that hyperinflammatory patients reflect a later stage of illness with more systemic dissemination. However, data on phenotype transitions over time suggest that temporal progression alone does not fully explain these phenotypes. Although the timing of infection onset is uncertain in human studies, longitudinal analyses from our group and others demonstrate that 94% to 98% of hypoinflammatory patients remain so over time rather than transitioning to hyperinflammatory.^28^^,^^29^ If hyperinflammatory patients were simply further along the same trajectory, a substantial proportion of hypoinflammatory patients would be expected to transition to hyperinflammatory over time, which has not been observed. These findings suggest that inflammatory phenotypes reflect, at least in part, intrinsic differences in host-pathogen interaction rather than position along a shared trajectory.

### Validation and clinical relevance in an in vivo mouse model

The pneumococcal pneumonia mouse model provides preclinical evidence for the inflammatory phenotypes observed in humans. More inflamed mice exhibited elevated plasma inflammatory biomarkers, greater bacterial loads, more severe lung injury, and lower survival than less inflamed mice. Importantly, plasma biomarkers measured at 18 hours postinfection reliably predicted 36-hour outcomes, including hypoxemia, BAL protein, and bacterial burden, underscoring the utility of early biomarker assessment for identifying phenotypes and predicting disease trajectory. The divergence in outcomes and biomarker profiles between phenotypes paralleled that observed in humans with hyperinflammatory vs hypoinflammatory pneumonia, ARDS, and sepsis,^8^^,^^10^^,^^14^ indicating that this model may be valuable for studying inflammatory response heterogeneity in acute lung injury, pneumonia, and sepsis. By modeling inflammatory phenotypes before therapeutic intervention, this translationally relevant model opens new possibilities for precision medicine, including testing candidate therapeutics for bacterial and viral pneumonia, the most common causes of ARDS.

A striking finding was the heterogeneity in inflammatory response and outcomes among genetically homogenous mice from the same vendor, raised under identical conditions, and given an identical infectious insult at a uniform time. This variability could not be explained by bacterial inoculum, as identical doses delivered a consistent pulmonary bacterial load, nor by batch effect. Several aspects of the design and findings argue against timing as a major driver of the observed differences between phenotypes. First, inoculation within each batch occurred in a narrow window (∼45 minutes), limiting temporal variability. Second, infection order within batches was not associated with phenotype assignment (Figure S7, see online supplementary material), arguing against systematic differences in disease stage. Third, more inflamed mice demonstrated early divergence in body temperature and plasma biomarkers before antibiotic initiation, indicating that phenotype separation occurs early rather than reflecting later progression along a shared trajectory. Finally, adjustment for baseline body weight did not materially alter the results, suggesting that differences in initial observable host characteristics do not explain phenotype assignment. Together, these findings support the interpretation that the observed classes reflect host-response patterns rather than different temporal stages of a common process. Although conditions were highly standardized, unmeasured factors such as immune priming, microbiota, circadian rhythms, social stress, and ambient exposures may contribute to interindividual differences in host response, consistent with recent histopathology-based subphenotyping of mouse pneumonia models.^30^

### Potential for phenotype-specific therapies

Our analysis revealed clear heterogeneity in response to both steroids and IL-6 receptor antagonists. Consistent with prior work, dexamethasone significantly reduced lung injury in mice overall,^31^^,^^32^ but this improvement was driven primarily by the more inflamed phenotype, in which dexamethasone increased oxygenation (SpO_2_) and reduced inflammatory biomarkers. In contrast, the less inflamed phenotype derived little benefit. These findings may reflect both prognostic and predictive enrichment. Although more inflamed mice exhibited greater physiological derangement, all animals were identical before infection, and adjustment for baseline observable differences (eg, body weight) did not materially alter the phenotype-by-treatment interaction. These results highlight the potential value of stratifying treatment by inflammatory phenotype, because treatment response varied markedly with the underlying inflammatory state, allowing the most appropriate intervention to be selected and avoiding treatments that might be ineffective or detrimental in certain phenotypes. Real-time plasma protein biomarker measurement is now feasible in patients,^33^^,^^34^ and a clinical trial has begun using real-time stratification by inflammatory phenotypes^35^ (www.panthertrial.org). These findings indicate that anti-inflammatory therapies like corticosteroids or IL-6 receptor antagonists may be good candidates for testing in phenotype-targeted trials and, more broadly, that animal models incorporating biological heterogeneity can screen candidates for precision therapy in critically ill patients.

### Strengths, limitations, and future directions

This study has several strengths, including the integration of prospective clinical data with experimental models, a clinically relevant mouse model with antibiotic treatment, and rigorously applied LCA procedures. It also has limitations. First, the absence of a clinical validation cohort may limit the generalizability, although strong concordance with prior ARDS and sepsis studies of more than 12 000 patients mitigates this concern.^8^^,^^10^^,^^14^ Second, the low rate of positive cultures limits our ability to link phenotypes to specific pathogens, although this rate mirrors that of high-quality epidemiologic studies of pneumonia;^36^ future metagenomic studies could help close this gap. Third, the mouse model, although translationally relevant, does not fully capture the complexity of human pneumonia or pulmonary sepsis, including multiorgan involvement and diverse pathogens;^37^ moreover, the mice correspond to an adolescent or young-adult stage, differing from the typically older clinical population in ways that may influence host-response and generalizability. Fourth, the mouse model recapitulates some aspects of the biological heterogeneity observed in humans but not all features of the hyperinflammatory and hypoinflammatory phenotypes. For example, Ang-2 and ICAM-1, consistently higher in plasma from hyperinflammatory than in hypoinflammatory patients, displayed the opposite pattern in mice. This discrepancy may reflect species-specific immune or endothelial responses,^38^ temporal dynamics of inflammation,^39^ or compartmentalization,^26^ underscoring the caution needed in cross-species extrapolation. Fifth, mouse phenotype classification used pretreatment biomarker data analyzed retrospectively, so treatment-response analyses were post hoc; although this ensured blinding during experiments and is consistent with all prior human studies, it limits prospective inference. Future work will develop a parsimonious classifier prospective application in mice, analogous to human approaches. As additional dimensions of biological heterogeneity (metabolomic, transcriptomic, microbial) are identified between phenotypes,^17^^,^^40^ animal models should be developed to incorporate them.

## Conclusions

These studies highlight the clinical and translational significance of inflammatory phenotypes in severe pneumonia and pulmonary sepsis. The identification of more and less inflamed phenotypes in mice, with their differing treatment responses, underscores the potential of phenotype-informed precision medicine. By integrating human clinical data with experimental findings in a clinically relevant mouse model, this study demonstrates the feasibility of phenotype-informed preclinical modeling for pneumonia, ARDS, and sepsis, providing a platform for future studies of phenotype-targeted therapies aimed at improving outcomes for critically ill patients.

## Supplementary Material

aamag402_Supplementary_Data

## Data Availability

All data associated with this study are available in the paper or the supplementary materials. All raw and processed data, including QA/QC documentation, are stored in a Box folder shared among study investigators. Access to the full data set and analysis files can be provided upon reasonable request. All requests for materials will be fulfilled by M.A.M or C.S.C. after completion of material transfer agreements. This article has an online data supplement, which is accessible from this issue’s table of contents online at www.atsjournals.org
