# Supplementary material for: Inflammatory phenotypes in severe pneumonia: clinical evidence to mouse models for precision therapeutics
Source: Am J Respir Crit Care Med. 2026 Jul 31;212(11):2792–802. doi: 10.1093/ajrccm/aamag402 (PMC13721131; doi:10.1093/ajrccm/aamag402)
Supplement: Supplementary file 1 [file aamag402_supplementary_data.zip › Phenotypes_supplementary text_R1_final.docx]

**Inflammatory Phenotypes in Severe Pneumonia: Clinical Evidence to Mouse Models for Precision Therapeutics**

**SUPPLEMENTARY MATERIALS**

## Supplementary figures:

[Figure S1: Human study Flowchart. Patients who had experienced cardiac arrest prior to ICU admission were excluded from the study, as the associated inflammatory response could confound the analysis.	14](#_Toc232147584)

[Figure S 2: Sensitivity analysis restricted to patients without ARDS. A. Mean values of physiological and inflammatory biomarkers across latent classes identified in patients without ARDS. B. ICU mortality according to latent class assignment.	15](#_Toc232147585)

[Figure S3: Mean estimates of physiological and inflammatory markers across latent class analyses in all mice (n=222, including mice from development phase and therapeutic test phase) and without Ang-2 and ICAM-1. Interleukin-8 measured as KC in the mice. Estimates are shown with their standard errors (error bars).	16](#_Toc232147586)

[Figure S4: Effect of dexamethasone on plasma biomarkers in mice. A. Evolution of plasma biomarkers measured at 18h and 36h among the control group and the group receiving dexamethasone. B. Evolution of plasma biomarkers measured at 18h and 36h among the control group and the group receiving dexamethasone and stratified by phenotype.	18](#_Toc232147587)

[Figure S5 : Effect of IL-6 receptor antagonist on plasma biomarkers in mice. A. Evolution of plasma biomarkers measured at 18h and 36h among the control group and the group receiving IL-6 receptor antibody. B. Evolution of plasma biomarkers measured at 18h and 36h among the control group and the group receiving IL-6 receptor antibody and stratified by phenotype.	20](#_Toc232147588)

[Figure S6: Heterogeneity among mice batches in physiological and inflammatory parameters. Density ridge plots depict the distribution of baseline body temperature (A), baseline body weight (B), Spo2 at 36h (E), total protein concentration in BAL at 36 hours (F) and IL-6 plasma concentration at 36h (G) across different experimental groups. (C) and (D), respectively displayed the proportion of deceased animals and the proportion of animals classified as belonging to the hyperinflammatory class in each batch.	22](#_Toc232147589)

[Figure S7: **Heatmap showing the chronological distribution of latent inflammatory classes across experimental infection batches.** Each tile represents an individual mouse ordered according to the chronological sequence of infection within each experimental batch. Rows correspond to experimental batches, and colors indicate latent class assignment derived from the latent class analysis.	24](#_Toc232147590)

[Figure S8: Bacterial Load in BAL 1 hour after intranasal inoculation with 2x10e8 S. pneumoniae.	25](#_Toc232147591)

[Figure S9: Sex-specific differences in physiological responses and lung injury in mice with pneumococcal pneumonia. A. Evolution of physiological responses following intranasal inoculation with Streptococcus pneumoniae, comparing female (red) and male (blue) mice. B. Indices of lung injury measured at 36 hours after bacterial inoculation. Each dot represents an individual mouse, and horizontal bars indicate group means ± standard deviation. No apparent differences in physiological responses or lung injury were observed between female and male mice.	26](#_Toc232147592)

[Figure S10: Evolution of arterial hypoxemia as measured by oxygen saturation (SpO₂) (A) and body temperature (B) over time in mice. Boxplots represent the distribution of observed body temperatures or SpO2 at each time point. The color of the boxplots corresponds to the median body temperature or SpO2, following a gradient from blue (lower values) to red (higher values).	27](#_Toc232147593)

[Figure S11: A. Scatter plot illustrating the relationship between PaO_2_/FiO_2_ (left) and RAGE (right) with IL-6, and IL-8 in human data. The regression line (black) is shown with its 95% confidence interval (grey zone), along with the Spearman correlation coefficient. A color gradient from red to blue represents values of RAGE and the PaO₂/FiO₂ ratios. B. Correlation between biomarkers at 18h and arterial oxygen saturation (left) or Total proteins in BAL (right) in the mouse experiment. The scatter plots include a regression line (black) with a 95% confidence interval (gray shading). Colors indicate values of y-axis variables, ranging from red (higher) to blue (lower). All biomarker values are log-transformed. Spearman correlation coefficients were used to assess associations.	28](#_Toc232147594)

[Figure S12: Correlation between plasma biomarkers at 18 hours and arterial oxygen saturation (A), Total proteins in BAL (B), bacterial load in BAL at 36 hours (C) and body temperature (D) in mice. The scatter plots include a regression line (black) with a 95% confidence interval (gray shading). Colors indicate values of y-axis variables, ranging from red (higher) to blue (lower). All biomarker values are log-transformed. Spearman correlation coefficients were used to assess associations and p value were adjusted using false discovering rate correction for multiple testing.	29](#_Toc232147595)

[Figure S13: Correlation between plasma biomarkers at 36 hours and arterial oxygen saturation (A), Total proteins in BAL (B), bacterial load in BAL at 36 hours (C) and body temperature (D) in mice. The scatter plots include a regression line (black) with a 95% confidence interval (gray shading). Colors indicate values of y-axis variables, ranging from red (higher) to blue (lower). All biomarker values are log-transformed. Spearman correlation coefficients were used to assess associations and p value were adjusted using false discovering rate correction for multiple testing.	31](#_Toc232147596)

[Figure S14: Correlation between plasma biomarkers and BAL biomarkers at 36 hours in mice. This figure illustrates the correlation between IL-6 (A) and KC (B) in plasma and BAL at 36 hours in the mouse control group. The scatter plots include a regression line (black) with a 95% confidence interval (gray shading). Colors indicate values, ranging from blue to red.	33](#_Toc232147597)

## Supplementary tables

[Table S1: Variables used for the latent class analysis in patients with pulmonary sepsis	34](#_Toc232147598)

[Table S2: Additional characteristics of patients with pulmonary sepsis stratified by phenotype	35](#_Toc232147599)

[Table S3. Results of the Latent Class Analysis in 548 patients with pulmonary sepsis	37](#_Toc232147600)

[Table S4: Results of the Latent Class Analysis excluding patient with ARDS	38](#_Toc232147601)

[Table S5. Performance of the parsimonious model with 4 and 3 variables with a cutoff of 0.5 in the pneumonia patients	39](#_Toc232147602)

[Table S6. Microbiology identification in blood culture, respiratory culture and viral detection stratified by inflammatory phenotype in n=548 pneumonia patients	40](#_Toc232147603)

[Table S7: Results of the Latent Class Analysis in the Mice Development Phase Model of Pneumonia (N=107)	42](#_Toc232147604)

[Table S8. Sensitivity analysis of the Latent Class Analysis in the Mice Development Phase Model of Pneumonia (N=107), including Ang-2 and ICAM-1	43](#_Toc232147605)

[Table S9. Contingency table comparing LCA classification from the initial development phase model and the sensitivity analysis including Ang-2 and ICAM-1.	44](#_Toc232147606)

[Table S10: Clinical and biological characteristics of mice infected with S. pneumoniae at baseline, 18h and 36h post-infection, stratified by phenotype	45](#_Toc232147607)

[Table S11: Results of the Latent Class Analysis in all Mice (Development model and therapeutic model) (N=222).	47](#_Toc232147608)

[Table S12: Sensitivity analysis of the Latent Class Analysis in the Therapeutic Model of Pneumonia (N=222), adding Ang-2 and ICAM-1 as biomarker	48](#_Toc232147609)

[Table S13: Contingency table comparing LCA classification from the initial development phase model and the sensitivity model including Ang-2 and ICAM-1	49](#_Toc232147610)

[Table S14: Characteristics of groups before randomization in the Dexamethasone experiments	50](#_Toc232147611)

[Table S15: Comparison of treatment effect (dexamethasone) at 36 hours stratified by class	52](#_Toc232147612)

[Table S16: Characteristics of groups before randomization in the IL-6 RA experiment	54](#_Toc232147613)

[Table S17: Comparison of treatment effect (IL-6 RA) at 36 hours stratified by class	56](#_Toc232147614)

[Table S 18: Interaction model for the dexamethasone experiment adjusted for baseline body weight	59](#_Toc232147615)

[Table S 19: Interaction model for the IL6-RA experiment adjusted for baseline body weight	60](#_Toc232147616)

[Table S 20: Mixed-effect model for the dexamethasone experiment incorporating batch as a random effect	61](#_Toc232147617)

[Table S 21: Mixed-effect model for the IL6-RA experiment incorporating batch as a random effect	62](#_Toc232147618)

**Supplementary Methods**

***Human study subjects and data collection***

Patients who had experienced cardiac arrest prior to ICU admission were excluded from the study, as the associated inflammatory response could confound the analysis

***Latent class analysis in patients***

Plasma biomarker concentrations were measured on samples taken at the time of enrollment, as previously described (1). Briefly, blood samples were processed and plasma stored at -80°C. until batch quantification. Plasma samples frozen at baseline were thawed for protein biomarker quantification. Samples were frozen in small aliquots to minimize freeze-thaw cycles. Singleplex assays were used for the quantification of protein C (Helena Laboratories; Beaumont, TX) and plasminogen activator inhibitor-1 (PAI-1, R&D systems; Minneapolis, MN) and were measured as duplicates. All other biomarkers-- interleukin-8 (IL-8), interleukin-6 (IL- 6), soluble tumor-necrosis factor receptor-1 (sTNFR-1), Angiopoietin-2 (Ang-2), and intercellular adhesion molecule-1 (ICAM-1) -- were measured using a multiplex ELISA (R&D systems; Minneapolis, MN). All multiplex assays were performed as single quantifications. For all biomarkers, a thorough quality control procedure was followed for each analyzed plate. Latent class analysis (LCA) of baseline clinical data and baseline plasma biomarker data was conducted using MPlus software. Nineteen variables were used to define the classes including, age, temperature, heart rate, systolic blood pressure, respiratory rate, hematocrit, white blood cell count, platelets, sodium, glucose, creatinine, bicarbonate, Interleukin (IL)-6, IL-8, Tumor Necrosis Factor Receptor-1 (TNFR-1), InterCellular Adhesion Molecule 1 (ICAM-1), Protein C, PAI-1, and Angiopoietin 2 (Ang-2). These variables were selected to be similar to those used in prior studies of the hyperinflammatory and hypoinflammatory phenotypes (1-4). As previously described, no clinical outcomes were included in the modeling process (1-4). Prior to modeling, biomarker values were carefully processed. When levels exceeded the upper limit of detection, they were replaced by the maximum measurable value. Values below the lower limit of detection were replaced by half that limit. All continuous variables were standardized using log transformation and z-scoring. Then, we assessed the correlations among biomarkers using Spearman correlation coefficients. When two variables showed a correlation coefficient greater than 0.5, their correlation was explicitly added inside the LCA model (5).

Four models consisting of one to four classes were constructed, and the best-fitting model was selected based on multiple criteria (5), including the Bayesian Information Criterion (BIC), the Vuong-Lo Mendell-Rubin (VLMR) likelihood ratio test, entropy, and the size of the smallest class. Once the optimal model was identified, each patient was assigned to a class based on the highest posterior probability.

***Parsimonious classifier models***

We compared the results from the LCA to two previously developed and validated biomarker-based parsimonious classifier models (PCM). Theses PCMs were initially developed on multiple cohorts to classify ARDS phenotypes and rely on research plasma biomarkers such as IL-8, protein C, sTNFR-1, serum bicarbonate, and vasopressor use (6). We compared the PCMs classification using a default probability cutoff of 0.5 to the gold standard of LCA-derived class assignment using the area under receiver-operating characteristic curves. Metrics such as sensitivity, specificity, precision, and AUC were calculated to assess the model.

***Microbiology assessment***

Clinical microbiological data were derived from clinician-ordered tests collected within the first 48 hours. For bacterial and fungal pathogens, these included blood and/or lower respiratory tract (tracheal aspirate or bronchoalveolar lavage, BAL) cultures. Viral infections were assessed by nasopharyngeal or lower respiratory samples using multiplex PCR. Microbiology results were systematically reviewed by ≥2 physicians to ensure only established pneumonia pathogens were included and common skin or environmental contaminants were excluded (7). In case of positive blood culture, the pathogen isolated from blood was prioritized

***Mouse experiments***

The mouse experiments were conducted using a well-characterized model of pneumococcal pneumonia in female C57BL/6 mice aged 9–12 weeks. All mice received antibiotic treatment to reflect standard clinical care. The sample size was determined in an exploratory manner, as the primary aim was to identify and characterize inflammatory phenotypes rather than to test a predefined hypothesis. For therapeutic studies, mice were randomly assigned to intervention or control groups and treated with either dexamethasone, an IL-6 receptor antibody, or vehicle control. Sample collection and outcome assessment were conducted at predefined time points. Detailed protocols are provided in the following sections.

***Latent class analysis in mice***

Biomarkers (IL-6, KC, TNFR-1, and MMP-8, Ang-2 and ICAM-1) were measured in plasma collected at 18 hours post-infection. Blood samples (100 µL) were obtained via submandibular vein puncture. The biomarkers were measured using the FLEXMAP 3DTM system (Luminex) with a Mouse Luminex Discovery Assay kit (R&D Systems). The timing of biomarker collection at 18 hours post-infection was chosen because this corresponds to the onset of hypoxemia as reflected in the decline of SpO₂ decline (8), a critical indicator of disease progression and the degree of pulmonary edema (8). These 18h samples were collected prior to any therapeutic intervention.

To conduct the LCA, we constructed four models containing one to four latent classes. As in the human data, the optimal model was selected based on a combination of the Bayesian Information Criterion (BIC), the Vuong-Lo Mendell-Rubin Likelihood Ratio Test (VLMR test), entropy, and the size of the smallest class (27). Once the best-fitting model was identified, each mouse was assigned to a class membership based on the highest posterior probability.

***Assessment of lung injury and bacterial load***

Bronchoalveolar lavage (BAL) was performed after exsanguination to evaluate protein concentration, total cell counts, and bacterial titers. The trachea was cannulated, and the lungs were lavaged twice with 250 µL of sterile PBS. Protein concentration in the BAL fluid was measured using the BCA Protein Assay (Thermo Fisher Scientific, Waltham, MA). The total BAL cell count was determined using a Coulter counter. Bacterial titers measured in colony forming units per mL were assessed by serial dilutions and plating on sheep blood agar.

***Therapeutic interventions in experimental mice***

Therapeutic interventions were conducted in the pneumococcal pneumonia mouse model to assess the effects of treatments that are clinically relevant but whose efficacy remains a subject of ongoing debate in clinical settings. At 18 hours post-inoculation, mice were randomized into intervention or control groups. The intervention groups received either dexamethasone (10 mg/kg) or an IL-6 receptor antibody (2 mg/mouse) combined with ceftriaxone (150 mg/kg) via intraperitoneal injection. The control groups received ceftriaxone plus vehicle control. Both dexamethasone and IL-6 receptor antibody showed efficacy in COVID-19 and dexamethasone has been tested in ARDS and sepsis with mixed results in different trials (9-11). Mice were monitored for body temperature, body weight, and oxygen saturation (SpO₂) at baseline and at 18, and 36 hours post-inoculation. Mice were sacrificed 36 hours post-inoculation, and outcomes, including lung injury and bacterial load, were assessed to evaluate the efficacy of the therapeutic interventions and their impact on different phenotypes.

**Male mice**. Although female mice were used for these studies because of difficulties with male mice co-existing together, in subsequent studies we were able to test male mice which did have the same responses to the bacterial pneumonia as the female mice *(****Figure S8).***

***Additional analyses for potential confounding by disease stage***

To assess whether differences in phenotype assignment could be explained by variation in disease stage, we performed additional analyses in the murine cohort. Baseline body weight was included as a covariate in sensitivity analyses to account for its potential influence on susceptibility to pneumococcal infection.

**Supplementary Results**

***Model selection for latent class analysis (Patients)***

Latent class models with one to four classes were evaluated to determine the optimal number of classes in the pneumonia cohort. The Vuong–Lo–Mendell–Rubin (VLMR) likelihood ratio test comparing the two-class model to the one-class model was statistically significant (p = 0.0008). Entropy values indicated adequate class separation across all tested models. The Bayesian Information Criterion (BIC) demonstrated the greatest reduction between the one-class and two-class models (***Table S3***). Although models with more than two classes showed modest additional improvement in BIC and the VLMR test remained significant for both the two- and three-class solutions, the three-class model included one class representing less than 5% of the cohort. This raised concerns regarding clinical interpretability and model stability. Accordingly, the two-class model was selected for all subsequent analyses.

***Parsimonious Classifier Models in the Patients***

We tested whether the classes (phenotypes) identified by LCA in patients with pulmonary sepsis corresponded to the hyperinflammatory and hypoinflammatory phenotypes previously described in ARDS and sepsis. To do so, we used two parsimonious classifier models that were previously trained in over 2000 patients to identify the hyperinflammatory phenotype in ARDS (6). Phenotype classification using these two models was highly concordant with the LCA-derived classes, with area under the receiver-operator curve (AUC) of 0.97 and 0.96 for the 4-variables and 3- variables models respectively, compared to the gold standard of LCA-assigned class ***(Table S5).*** This finding indicates that the phenotypes identified in this cohort of patients with pulmonary sepsis are analogous to those phenotypes previously identified in ARDS/sepsis, providing further evidence that this phenotyping schema is conserved across these overlapping syndromes in critical illness.

***Microbiological Data in the Patients***

To provide context for the translational alignment between the human cohort and the mouse model using a single pathogen (*Streptococcus pneumoniae*), we examined microbiological data in the patient cohort. Differences in pathogen distribution across phenotypes may provide insight into the biological drivers of the systemic inflammatory response and help place the findings from the single-pathogen model into a broader clinical context. Patients classified as hyperinflammatory were more likely to have positive blood cultures compared to those in the hypoinflammatory phenotype (37% versus 9%, p<0.001 respectively, ***Table S6***). Of blood culture pathogens, *Streptococcus pneumoniae* was more frequently isolated in hyperinflammatory patients compared to hypoinflammatory patients (14% versus 2%, p<0.001, respectively), followed by *Staphylococcus aureus* and *Escherichia coli*. Similarly, the identification of pathogens in respiratory cultures was more frequent in hyperinflammatory patients, with a higher proportion of *S. pneumoniae* and *E. coli* in respiratory cultures from the hyperinflammatory phenotype.

***Plasma Inflammatory Protein Biomarkers Correlate with the Severity of Lung Injury in Mice with Bacterial Pneumonia and in Humans with Pulmonary sepsis***

After having confirmed that the hyperinflammatory and hypoinflammatory phenotypes were observable in patients with pulmonary sepsis, we next asked whether the biomarkers which characterize these phenotypes were predictive of outcomes in our well-established mouse model of bacterial pneumonia. 107 mice were initially studied to test the association of plasma biomarkers and prognosis ***(Figure 1B)***. Mice were intranasally inoculated with *S. pneumoniae* and developed mild hypoxemia 18 hours post-infection, with the mean oxygen saturation (SpO_2_) decreasing to 92 ± 4% (***Figure S10A***). Mice were treated with ceftriaxone at 18 and 30 hours post-infection. Despite antibiotic treatment, arterial hypoxemia worsened, reaching a mean of 81 ± 11% at 36 hours post-infection (***Figure S10A***). The body temperature of the mice dropped at 12 hours post-infection, slightly increased at 18 hours, and remained stable through 36 hours (***Figure S10B***).

Despite a uniform infectious stimulus (2x10^8^ S. *pneumoniae* administered intranasally) in all mice and a uniform bacterial load delivered to the airspaces at 1 hour post-infection (see details below under ***Heterogeneity Across Batches***), the inflammatory response in the plasma was variable, with 2-3 log fold variance in the plasma levels of inflammatory biomarkers at 18 hours (***Figure S11-12***). Higher plasma inflammatory biomarkers at 18 hours post-infection, including IL-6, KC (the mouse orthologue for IL-8), MMP-8, and sTNFR-1, correlated with more severe metrics of acute lung injury at 36 hours, including lower SpO_2_, higher total protein concentration in the BAL (an indicator of alveolar permeability), higher BAL bacterial load, and lower body temperature (***Figure S11-12***). IL-6 and KC measured at 36 hours post-infection were also strongly correlated with SpO_2_, total protein concentration in BAL, and bacterial load in the air spaces (***Figure S13***). Additionally, IL-6 and KC measured at 36 hours in plasma demonstrated a strong correlation with IL-6 and KC measured in BAL (***Figure S14***).

In patients with pneumonia, IL-6 and IL-8 were similarly correlated with hypoxemia, as measured by the PaO_2_/FiO_2_ ratio (Spearman coefficient: -0.35 and -0.32 respectively, p<0.001 for both; ***Figure S11***), and with plasma sRAGE levels, an indicator of alveolar epithelial lung injury (Spearman coefficient: 0.47 and 0.43 respectively, p<0.001 for both).

***Model selection for latent class analysis (Mice)***

Model fit indices supported a two-class solution in the mouse pneumonia cohort. Entropy values indicated adequate class separation across all tested models. The Bayesian Information Criterion (BIC) showed the greatest reduction between the one-class and two-class models (***Table S7***). Models with more than two classes were associated with higher BIC values, suggesting potential model overfitting, and the Vuong–Lo–Mendell–Rubin (VLMR) likelihood ratio test was not statistically significant for these models. Accordingly, the two-class model was selected as the best-fitting solution.

In this model, the “more inflamed” phenotype was characterized by higher levels of inflammatory biomarkers compared with the “less inflamed” phenotype, mirroring the hyperinflammatory phenotype observed in human pneumonia (***Figure 3A and B***), and by a higher incidence of bacteremia (100% versus 27%, p < 0.001).

Sensitivity analyses incorporating Ang-2 and ICAM-1 yielded similar class structures, with only minor differences in class assignment compared with the reference model (***Tables S8*** and ***S9***). Based on these findings, the primary model was retained for all subsequent analyses.

***Biomarker responses in the therapeutic experiments***

Additional biomarker and lung injury outcomes from the dexamethasone experiment are shown in ***Figure S4*** and ***Table S15***. Additional biomarker and lung injury outcomes from the IL-6 receptor antibody experiment are shown in ***Figure S5*** and ***Table S17***.

***Additional analyses addressing potential differences in disease stage (Mice)***

To address the possibility that the identified classes reflect differences in disease stage rather than distinct host-response patterns, we performed several additional analyses.

First, we examined whether infection order within each batch (used as a proxy for small differences in infection timing) was associated with phenotype assignment (***Figure S8***). There was no evidence of a systematic relationship between mouse order and class membership, suggesting that minor differences in infection timing did not drive phenotype classification.

Second, we evaluated the potential influence of baseline body weight, given prior evidence that body weight may affect susceptibility to pneumococcal infection (***Table S18-19***). Adjustment for baseline body weight did not materially alter associations between phenotype and outcomes.

Third, consistent with our primary results, we observed early divergence between classes in physiological and inflammatory markers prior to antibiotic administration, including differences in SpO₂, body temperature, and plasma biomarkers (***Table S10***). These findings support the interpretation that class separation occurs early in the disease course rather than reflecting later stages along a common trajectory.

Together, these analyses suggest that the identified classes are unlikely to be explained solely by differences in disease stage at a fixed time point.
