# Supplementary material for: Inflammatory phenotypes in severe pneumonia: clinical evidence to mouse models for precision therapeutics
Source: Am J Respir Crit Care Med. 2026 Jul 31;212(11):2792–802. doi: 10.1093/ajrccm/aamag402 (PMC13721131; doi:10.1093/ajrccm/aamag402)
Supplement: Supplementary file 1 [file aamag402_supplementary_data.zip › Phenotypes_supplementary figures&tables_R1_final.docx]

**Inflammatory Phenotypes in Severe Pneumonia: Clinical Evidence to Mouse Models for Precision Therapeutics**

**SUPPLEMENTARY Figures & Tables**

## Supplementary figures:

[Figure S1: Human study Flowchart. Patients who had experienced cardiac arrest prior to ICU admission were excluded from the study, as the associated inflammatory response could confound the analysis.	14](#_Toc232147584)

[Figure S 2: Sensitivity analysis restricted to patients without ARDS. A. Mean values of physiological and inflammatory biomarkers across latent classes identified in patients without ARDS. B. ICU mortality according to latent class assignment.	15](#_Toc232147585)

[Figure S3: Mean estimates of physiological and inflammatory markers across latent class analyses in all mice (n=222, including mice from development phase and therapeutic test phase) and without Ang-2 and ICAM-1. Interleukin-8 measured as KC in the mice. Estimates are shown with their standard errors (error bars).	16](#_Toc232147586)

[Figure S4: Effect of dexamethasone on plasma biomarkers in mice. A. Evolution of plasma biomarkers measured at 18h and 36h among the control group and the group receiving dexamethasone. B. Evolution of plasma biomarkers measured at 18h and 36h among the control group and the group receiving dexamethasone and stratified by phenotype.	18](#_Toc232147587)

[Figure S5 : Effect of IL-6 receptor antagonist on plasma biomarkers in mice. A. Evolution of plasma biomarkers measured at 18h and 36h among the control group and the group receiving IL-6 receptor antibody. B. Evolution of plasma biomarkers measured at 18h and 36h among the control group and the group receiving IL-6 receptor antibody and stratified by phenotype.	20](#_Toc232147588)

[Figure S6: Heterogeneity among mice batches in physiological and inflammatory parameters. Density ridge plots depict the distribution of baseline body temperature (A), baseline body weight (B), Spo2 at 36h (E), total protein concentration in BAL at 36 hours (F) and IL-6 plasma concentration at 36h (G) across different experimental groups. (C) and (D), respectively displayed the proportion of deceased animals and the proportion of animals classified as belonging to the hyperinflammatory class in each batch.	22](#_Toc232147589)

[Figure S7: **Heatmap showing the chronological distribution of latent inflammatory classes across experimental infection batches.** Each tile represents an individual mouse ordered according to the chronological sequence of infection within each experimental batch. Rows correspond to experimental batches, and colors indicate latent class assignment derived from the latent class analysis.	24](#_Toc232147590)

[Figure S8: Bacterial Load in BAL 1 hour after intranasal inoculation with 2x10e8 S. pneumoniae.	25](#_Toc232147591)

[Figure S9: Sex-specific differences in physiological responses and lung injury in mice with pneumococcal pneumonia. A. Evolution of physiological responses following intranasal inoculation with Streptococcus pneumoniae, comparing female (red) and male (blue) mice. B. Indices of lung injury measured at 36 hours after bacterial inoculation. Each dot represents an individual mouse, and horizontal bars indicate group means ± standard deviation. No apparent differences in physiological responses or lung injury were observed between female and male mice.	26](#_Toc232147592)

[Figure S10: Evolution of arterial hypoxemia as measured by oxygen saturation (SpO₂) (A) and body temperature (B) over time in mice. Boxplots represent the distribution of observed body temperatures or SpO2 at each time point. The color of the boxplots corresponds to the median body temperature or SpO2, following a gradient from blue (lower values) to red (higher values).	27](#_Toc232147593)

[Figure S11: A. Scatter plot illustrating the relationship between PaO_2_/FiO_2_ (left) and RAGE (right) with IL-6, and IL-8 in human data. The regression line (black) is shown with its 95% confidence interval (grey zone), along with the Spearman correlation coefficient. A color gradient from red to blue represents values of RAGE and the PaO₂/FiO₂ ratios. B. Correlation between biomarkers at 18h and arterial oxygen saturation (left) or Total proteins in BAL (right) in the mouse experiment. The scatter plots include a regression line (black) with a 95% confidence interval (gray shading). Colors indicate values of y-axis variables, ranging from red (higher) to blue (lower). All biomarker values are log-transformed. Spearman correlation coefficients were used to assess associations.	28](#_Toc232147594)

[Figure S12: Correlation between plasma biomarkers at 18 hours and arterial oxygen saturation (A), Total proteins in BAL (B), bacterial load in BAL at 36 hours (C) and body temperature (D) in mice. The scatter plots include a regression line (black) with a 95% confidence interval (gray shading). Colors indicate values of y-axis variables, ranging from red (higher) to blue (lower). All biomarker values are log-transformed. Spearman correlation coefficients were used to assess associations and p value were adjusted using false discovering rate correction for multiple testing.	29](#_Toc232147595)

[Figure S13: Correlation between plasma biomarkers at 36 hours and arterial oxygen saturation (A), Total proteins in BAL (B), bacterial load in BAL at 36 hours (C) and body temperature (D) in mice. The scatter plots include a regression line (black) with a 95% confidence interval (gray shading). Colors indicate values of y-axis variables, ranging from red (higher) to blue (lower). All biomarker values are log-transformed. Spearman correlation coefficients were used to assess associations and p value were adjusted using false discovering rate correction for multiple testing.	31](#_Toc232147596)

[Figure S14: Correlation between plasma biomarkers and BAL biomarkers at 36 hours in mice. This figure illustrates the correlation between IL-6 (A) and KC (B) in plasma and BAL at 36 hours in the mouse control group. The scatter plots include a regression line (black) with a 95% confidence interval (gray shading). Colors indicate values, ranging from blue to red.	33](#_Toc232147597)

## Supplementary tables

[Table S1: Variables used for the latent class analysis in patients with pulmonary sepsis	34](#_Toc232147598)

[Table S2: Additional characteristics of patients with pulmonary sepsis stratified by phenotype	35](#_Toc232147599)

[Table S3. Results of the Latent Class Analysis in 548 patients with pulmonary sepsis	37](#_Toc232147600)

[Table S4: Results of the Latent Class Analysis excluding patient with ARDS	38](#_Toc232147601)

[Table S5. Performance of the parsimonious model with 4 and 3 variables with a cutoff of 0.5 in the pneumonia patients	39](#_Toc232147602)

[Table S6. Microbiology identification in blood culture, respiratory culture and viral detection stratified by inflammatory phenotype in n=548 pneumonia patients	40](#_Toc232147603)

[Table S7: Results of the Latent Class Analysis in the Mice Development Phase Model of Pneumonia (N=107)	42](#_Toc232147604)

[Table S8. Sensitivity analysis of the Latent Class Analysis in the Mice Development Phase Model of Pneumonia (N=107), including Ang-2 and ICAM-1	43](#_Toc232147605)

[Table S9. Contingency table comparing LCA classification from the initial development phase model and the sensitivity analysis including Ang-2 and ICAM-1.	44](#_Toc232147606)

[Table S10: Clinical and biological characteristics of mice infected with S. pneumoniae at baseline, 18h and 36h post-infection, stratified by phenotype	45](#_Toc232147607)

[Table S11: Results of the Latent Class Analysis in all Mice (Development model and therapeutic model) (N=222).	47](#_Toc232147608)

[Table S12: Sensitivity analysis of the Latent Class Analysis in the Therapeutic Model of Pneumonia (N=222), adding Ang-2 and ICAM-1 as biomarker	48](#_Toc232147609)

[Table S13: Contingency table comparing LCA classification from the initial development phase model and the sensitivity model including Ang-2 and ICAM-1	49](#_Toc232147610)

[Table S14: Characteristics of groups before randomization in the Dexamethasone experiments	50](#_Toc232147611)

[Table S15: Comparison of treatment effect (dexamethasone) at 36 hours stratified by class	52](#_Toc232147612)

[Table S16: Characteristics of groups before randomization in the IL-6 RA experiment	54](#_Toc232147613)

[Table S17: Comparison of treatment effect (IL-6 RA) at 36 hours stratified by class	56](#_Toc232147614)

[Table S 18: Interaction model for the dexamethasone experiment adjusted for baseline body weight	59](#_Toc232147615)

[Table S 19: Interaction model for the IL6-RA experiment adjusted for baseline body weight	60](#_Toc232147616)

[Table S 20: Mixed-effect model for the dexamethasone experiment incorporating batch as a random effect	61](#_Toc232147617)

[Table S 21: Mixed-effect model for the IL6-RA experiment incorporating batch as a random effect	62](#_Toc232147618)

**Supplementary Figures**

## Supplementary Figures

Figure S1: Human study Flowchart. Patients who had experienced cardiac arrest prior to ICU admission were excluded from the study, as the associated inflammatory response could confound the analysis.

Figure S 2: ****Sensitivity analysis restricted to patients without ARDS.**** A. Mean values of physiological and inflammatory biomarkers across latent classes identified in patients without ARDS. B. ICU mortality according to latent class assignment.

Figure S3: Mean estimates of physiological and inflammatory markers across latent class analyses in all mice (n=222, including mice from development phase and therapeutic test phase) and without Ang-2 and ICAM-1. Interleukin-8 measured as KC in the mice. Estimates are shown with their standard errors (error bars).

*Abbreviations: IL-6: interleukin-6; KC: keratinocyte-derived chemokine; MMP-8: matrix metalloproteinase-8; TNFR-1: tumor necrosis factor receptor 1*

Figure S4: Effect of dexamethasone on plasma biomarkers in mice. A. Evolution of plasma biomarkers measured at 18h and 36h among the control group and the group receiving dexamethasone. B. Evolution of plasma biomarkers measured at 18h and 36h among the control group and the group receiving dexamethasone and stratified by phenotype.

*Abbreviations: ANG-2: angiopoietin-2; ICAM-1: intercellular adhesion molecule 1; IL-6: interleukin-6; KC: keratinocyte-derived chemokine; MMP-8: matrix metalloproteinase-8; TNFR-1: tumor necrosis factor receptor 1*

Figure S5 : Effect of IL-6 receptor antagonist on plasma biomarkers in mice. A. Evolution of plasma biomarkers measured at 18h and 36h among the control group and the group receiving IL-6 receptor antibody. B. Evolution of plasma biomarkers measured at 18h and 36h among the control group and the group receiving IL-6 receptor antibody and stratified by phenotype.

*Abbreviations: ANG-2: angiopoietin-2; ICAM-1: intercellular adhesion molecule 1; IL-6: interleukin-6; KC: keratinocyte-derived chemokine; MMP-8: matrix metalloproteinase-8; TNFR-1: tumor necrosis factor receptor 1*

Figure S6: Heterogeneity among mice batches in physiological and inflammatory parameters. Density ridge plots depict the distribution of baseline body temperature (A), baseline body weight (B), Spo2 at 36h (E), total protein concentration in BAL at 36 hours (F) and IL-6 plasma concentration at 36h (G) across different experimental groups. (C) and (D), respectively displayed the proportion of deceased animals and the proportion of animals classified as belonging to the hyperinflammatory class in each batch.

Figure S7: **Heatmap showing the chronological distribution of latent inflammatory classes across experimental infection batches.** Each tile represents an individual mouse ordered according to the chronological sequence of infection within each experimental batch. Rows correspond to experimental batches, and colors indicate latent class assignment derived from the latent class analysis.

Figure S8: Bacterial Load in BAL 1 hour after intranasal inoculation with 2x10e8 S. pneumoniae.

*Abbreviations: BAL: bronchoalveolar lavage; CFU: colony forming unit.*

Figure S9: Sex-specific differences in physiological responses and lung injury in mice with pneumococcal pneumonia. A. Evolution of physiological responses following intranasal inoculation with *Streptococcus pneumoniae*, comparing female (red) and male (blue) mice. B. Indices of lung injury measured at 36 hours after bacterial inoculation. Each dot represents an individual mouse, and horizontal bars indicate group means ± standard deviation. No apparent differences in physiological responses or lung injury were observed between female and male mice.

*Abbreviations: BAL: bronchoalveolar lavage; CFU: colony forming unit*

Figure S10: Evolution of arterial hypoxemia as measured by oxygen saturation (SpO₂) (A) and body temperature (B) over time in mice. Boxplots represent the distribution of observed body temperatures or SpO2 at each time point. The color of the boxplots corresponds to the median body temperature or SpO2, following a gradient from blue (lower values) to red (higher values).

Figure S11: A. Scatter plot illustrating the relationship between PaO_2_/FiO_2_ (left) and RAGE (right) with IL-6, and IL-8 in human data. The regression line (black) is shown with its 95% confidence interval (grey zone), along with the Spearman correlation coefficient. A color gradient from red to blue represents values of RAGE and the PaO₂/FiO₂ ratios. B. Correlation between biomarkers at 18h and arterial oxygen saturation (left) or Total proteins in BAL (right) in the mouse experiment. The scatter plots include a regression line (black) with a 95% confidence interval (gray shading). Colors indicate values of y-axis variables, ranging from red (higher) to blue (lower). All biomarker values are log-transformed. Spearman correlation coefficients were used to assess associations.

Figure S12: Correlation between plasma biomarkers at 18 hours and arterial oxygen saturation (A), Total proteins in BAL (B), bacterial load in BAL at 36 hours (C) and body temperature (D) in mice. The scatter plots include a regression line (black) with a 95% confidence interval (gray shading). Colors indicate values of y-axis variables, ranging from red (higher) to blue (lower). All biomarker values are log-transformed. Spearman correlation coefficients were used to assess associations and p value were adjusted using false discovering rate correction for multiple testing.

*Abbreviations: BAL: bronchoalveolar lavage; CFU: colony-forming unit; FDR: false discovering rate; IL-6: interleukin-6; KC: keratinocyte-derived chemokine; MMP-8: matrix metalloproteinase-8; TNFR-1: tumor necrosis factor receptor 1*

Figure S13: Correlation between plasma biomarkers at 36 hours and arterial oxygen saturation (A), Total proteins in BAL (B), bacterial load in BAL at 36 hours (C) and body temperature (D) in mice. The scatter plots include a regression line (black) with a 95% confidence interval (gray shading). Colors indicate values of y-axis variables, ranging from red (higher) to blue (lower). All biomarker values are log-transformed. Spearman correlation coefficients were used to assess associations and p value were adjusted using false discovering rate correction for multiple testing.

*Abbreviations: BAL: bronchoalveolar lavage; CFU: colony-forming unit; FDR: false discovering rate; IL-6: interleukin-6; KC: keratinocyte-derived chemokine; MMP-8: matrix metalloproteinase-8; TNFR-1: tumor necrosis factor receptor 1*

Figure S14: Correlation between plasma biomarkers and BAL biomarkers at 36 hours in mice. This figure illustrates the correlation between IL-6 (A) and KC (B) in plasma and BAL at 36 hours in the mouse control group. The scatter plots include a regression line (black) with a 95% confidence interval (gray shading). Colors indicate values, ranging from blue to red.

*Abbreviations: BAL: bronchoalveolar lavage; IL-6: interleukin-6; KC: keratinocyte-derived chemokine*

Table S1: Variables used for the latent class analysis in patients with pulmonary sepsis

| Use of vasopressors (Y/N) | White blood cell count | IL-6 |
| --- | --- | --- |
| Age | **Platelets** | **TNFR-1** |
| Temperature | **Sodium** | **ICAM-1** |
| Heart rate | **Glucose** | **Protein C** |
| Systolic blood pressure | **Creatinine** | **PAI-1** |
| Respiratory rate | **Bicarbonate** | **Ang-2** |
| Hematocrit | **IL-8** |  |

**Abbreviations:** ANG-2: angiopoietin-2; ICAM-1: intercellular adhesion molecule 1; IL-6: interleukin-6; IL-8: interleukin-8; PAI-1: plasminogen activator inhibitor-1; TNFR-1: tumor necrosis factor receptor 1.

Table S2: Additional characteristics of patients with pulmonary sepsis stratified by phenotype

| Characteristic | Overall  N = 548 | Hypoinflammatory  N = 451 | Hyperinflammatory  N = 97 | p-value |
| --- | --- | --- | --- | --- |
| Coronary artery disease | 87 (16%) | 78 (17%) | 9 (9.3%) | 0.050 |
| Congestive heart failure | 154 (29%) | 131 (30%) | 23 (25%) | 0.4 |
| Hypertension | 230 (42%) | 199 (44%) | 31 (32%) | 0.028 |
| COPD | 176 (33%) | 159 (36%) | 17 (19%) | 0.001 |
| Home O2 use | 103 (20%) | 98 (23%) | 5 (6%) | <0.001 |
| Cirrhosis | 38 (7%) | 31 (7%) | 7 (7%) | 0.9 |
| Diabetes Mellitus | 146 (27%) | 122 (27%) | 24 (25%) | 0.6 |
| Solid tumor with metastases | 56 (10%) | 44 (9.9%) | 12 (13%) | 0.4 |
| Solid organ transplant | 17 (4%) | 15 (5%) | 2 (3%) | 0.7 |
| On immunosuppression | 50 (9%) | 37 (8%) | 13 (14%) | 0.11 |
| On chronic dialysis | 30 (6%) | 23 (6%) | 7 (8%) | 0.4 |
| CKD | 93 (17%) | 78 (17%) | 15 (15%) | 0.7 |
| SAPS II | 49 (36, 66) | 45 (34, 58) | 72 (55, 88) | <0.001 |
| Heart Rate (bpm) | 115 (100, 130) | 111 (97, 126) | 130 (118, 144) | <0.001 |
| Systolic blood pressure (mmHg) | 82 (72, 93) | 84 (75, 98) | 72 (63, 79) | <0.001 |
| Temperature (°C) | 37.6 (37.1, 38.4) | 37.5 (37.1, 38.2) | 38.4 (37.6, 39.1) | <0.001 |
| Respiratory rate (cpm) | 35 (28, 40) | 33 (27, 40) | 36 (35, 42) | <0.001 |
| Sodium (mmol/L) | 136 (133, 139) | 137 (133, 139) | 133 (130, 137) | <0.001 |
| Potassium (mmol/L) | 3.7 (3.4, 4.2) | 3.8 (3.4, 4.2) | 3.6 (3.2, 4.1) | 0.016 |
| pH | 7.30 (7.21, 7.38) | 7.31 (7.25, 7.39) | 7.21 (7.09, 7.30) | <0.001 |
| Bicarbonate (mmol/L) | 22.0 (18.0, 26.0) | 23.0 (20.0, 27.0) | 17.0 (13.0, 20.0) | <0.001 |
| Creatinine (mg/L) | 1.19 (0.80, 1.87) | 1.09 (0.76, 1.77) | 1.72 (1.20, 2.69) | <0.001 |
| Bilirubin (mg/L) | 0.9 (0.6, 1.4) | 0.8 (0.5, 1.2) | 1.2 (0.8, 1.9) | <0.001 |
| Albumin (g/L) | 2.4 (2.1, 2.9) | 2.6 (2.1, 3.0) | 2.2 (1.7, 2.5) | <0.001 |
| Glucose (g/dl) | 113 (92, 139) | 113 (94, 138) | 110 (76, 143) | 0.11 |
| White blood cells (thousands) | 10 (7, 15) | 11 (8, 15) | 5 (2, 10) | <0.001 |
| Hemoglobin (g/dL) | 10.1 (8.5, 11.7) | 10.3 (8.7, 12.0) | 9.0 (7.1, 10.8) | <0.001 |
| Hematocrit | 31 (27, 36) | 31 (27, 37) | 27 (23, 32) | <0.001 |
| Platelets (thousands) | 185 (122, 247) | 198 (139, 263) | 95 (31, 158) | <0.001 |
| PaCO2 (mmHg) | 41 (34, 52) | 42 (35, 53) | 37 (32, 46) | 0.002 |

**Abbreviations:** COPD: Chronic obstructive pulmonary disease; Home O₂: Home oxygen use; CKD: Chronic kidney disease; SAPS II: Simplified Acute Physiology Score II; PaCO₂: Partial pressure of arterial carb

Table S3. Results of the Latent Class Analysis in 548 patients with pulmonary sepsis

| **Number of Class** | **BIC** | **Entropy** | **N1** | **N2** | **N3** | **N4** | **P-value** |
| --- | --- | --- | --- | --- | --- | --- | --- |
| 1 | 28,284 | - | 548 | - | - | - | - |
| 2 | 27,605 | 0.93 | 451 | 97 | - | - | 0.0008 |
| 3 | 27,440 | 0.95 | 444 | 79 | 25 | - | 0.0167 |
| 4 | 27,285 | 0.96 | 439 | 79 | 25 | 5 | 0.1865 |

**Abbreviation:** BIC: Bayesian information criteria.

Table S4: Results of the Latent Class Analysis excluding patient with ARDS

| **Number of Class** | **BIC** | **Entropy** | **N1** | **N2** | **N3** | **N4** | **P-value** |
| --- | --- | --- | --- | --- | --- | --- | --- |
| 1 | 12900.35 | - | 239 | - | - | - | - |
| 2 | 12592.60 | 0.907 | 173 | 66 | - | - | 0.0556 |
| 3 | 12533.95 | 0.938 | 168 | 54 | 17 | - | 0.1730 |
| 4 | 12499.24 | 0.950 | 164 | 17 | 53 | 5 | 0.6767 |

Table S5. Performance of the parsimonious model with 4 and 3 variables with a cutoff of 0.5 in the pneumonia patients

| **Model** | **Accuracy** | **Sensitivity** | **Specificity** | **AUC** |
| --- | --- | --- | --- | --- |
| 3 Variables (IL-8, Bicarbonate, Protein C) | 0.88 | 0.88 | 0.88 | 0.96 |
| 4 Variables (IL-8, TNFR-1, Bicarbonate, Vasopressors) | 0.88 | 0.92 | 0.87 | 0.97 |

**Abbreviations: AUC: area under the curve**; IL-8: interleukin-8; TNFR-1: tumor necrosis factor receptor 1.

Table S6. Microbiology identification in blood culture, respiratory culture and viral detection stratified by inflammatory phenotype in n=548 pneumonia patients

| **Characteristic** | **Hypoinflammatory N = 451**^1^ | **Hyperinflammatory**  **N = 97**^1^ | **p-value**^2^ |
| --- | --- | --- | --- |
| ***Blood culture*** |  |  |  |
| Blood culture sent | 359 (80%) | 83 (86%) | 0.2 |
| No growth | 327 (91%) | 52 (63%) | <0.001 |
| Streptococcus pneumoniae | 6 (2%) | 12 (14%) | <0.001 |
| Escherichia coli | 3 (1%) | 4 (5%) | 0.026 |
| Staphylococcus aureus | 8 (2%) | 6 (7%) | 0.031 |
| Other Gram positive | 3 (2%) | 0 (0%) | >0.9 |
| Other Gram negative | 5 (2%) | 8 (11%) | <0.001 |
| ***Respiratory culture*** |  |  |  |
| ***Respiratory culture sent*** | 255 (57%) | 60 (62%) | 0.3 |
| No growth | 198 (78%) | 31 (52%) | <0.001 |
| Streptococcus pneumoniae | 7 (3%) | 5 (8%) | 0.057 |
| Escherichia coli | 3 (1%) | 6 (10%) | 0.002 |
| Staphylococcus aureus | 25 (10%) | 8 (15%) | 0.4 |
| Other Gram positive | 1 (0%) | 0 (0%) | 0.9 |
| Other Gram negative | 16 (7%) | 8(15%) | 0.10 |
| ***Virus*** |  |  |  |
| Viral testing sent | 186 (41%) | 38 (39%) | 0.7 |
| Positive viral detection | 57 (31%) | 17 (45%) | 0.092 |
| Isolated viral identification | 42 (81%) | 6 (38%) | 0.003 |
| Viral species |  |  | 0.2 |
| Influenza | 19 (10%) | 7 (18%) |  |
| Other than influenzae virus | 38 (20%) | 10 (26%) |  |
| Negative detection for viral infection | 129 (69%) | 21 (55%) |  |

***^1^* n (%)**

***^2^* Pearson’s Chi-squared test; Fisher’s exact test**

Table S7: Results of the Latent Class Analysis in the Mice Development Phase Model of Pneumonia (N=107)

| **Number of Classes** | **BIC** | **Entropy** | **N1** | **N2** | **N3** | **N4** | **P-value** |
| --- | --- | --- | --- | --- | --- | --- | --- |
| 1 | 1,518 | - | 107 |  |  |  | - |
| 2 | 1,501 | 0.77 | 59 | 48 |  |  | 0.0286 |
| 3 | 1,505 | 0.81 | 34 | 49 | 24 |  | 0.0939 |
| 4 | 1,520 | 0.84 | 27 | 31 | 1 | 48 | 0.0993 |

**Abbreviations:** BIC: Bayesian information criteria

Table S8. Sensitivity analysis of the Latent Class Analysis in the Mice Development Phase Model of Pneumonia (N=107), including Ang-2 and ICAM-1

| **Number of Classes** | **BIC** | **Entropy** | **N1** | **N2** | **N3** | **N4** | **P-value** |
| --- | --- | --- | --- | --- | --- | --- | --- |
| 1 | 2,123 | - | 107 |  |  |  | - |
| 2 | 2,094 | 0.8 | 57 | 50 |  |  | 0.0106 |
| 3 | 2,097 | 0.84 | 51 | 34 | 22 |  | 0.4651 |
| 4 | 2,111 | 0.89 | 32 | 48 | 2 | 25 | 0.3378 |

**Abbreviations:** BIC: Bayesian information criteria

Table S9. Contingency table comparing LCA classification from the initial development phase model and the sensitivity analysis including Ang-2 and ICAM-1.

|  | **Class 1 (Sensitivity analysis)** | **Class 2 (Sensitivity analysis)** |
| --- | --- | --- |
| **Class 1 (Primary model)** | 55 | 4 |
| **Class 2 (Primary model)** | 2 | 46 |

Table S10: Clinical and biological characteristics of mice infected with S. pneumoniae at baseline, 18h and 36h post-infection, stratified by phenotype

| **Characteristic** | **Less inflamed**  **N = 59^1^** | **More inflamed**  **N = 48^1^** | **Adjusted p-value^2^** |
| --- | --- | --- | --- |
| ***Data collected at Baseline*** |  |  |  |
| Body Temperature (°C) | 38.0 (37.8 – 38.3) | 38.0 (37.6 – 38.2) | 0.91 |
| Body Weight (g) | 20.8 (19.9 – 22.2) | 20.0 (19.4 – 20.6) | 0.002 |
| SpO_2_ (%) | 98 (98– 99) | 98 (98 – 99) | 0.47 |
| ***Data collected at 12h*** |  |  |  |
| Body Temperature (°C) | 31.0 (29.3-32.2) | 28.3 (27.3-29.0) | <0.001 |
| ***Data collected at 18h*** |  |  |  |
| Body Temperature (°C) | 34.8 (33.0 – 35.4) | 31.0 (29.5 – 32.3) | <0.001 |
| SpO_2_ (%) | 92 (89 – 94) | 92 (88 – 94) | 0.96 |
| Bacteremia (%) | 16 (27) | 48 (100) | <0.001 |
| KC in Plasma (pg/mL) | 5,201 (3,318 – 8,250) | 7,385 (5,258 – 11,108) | 0.040 |
| sTNFR1 in Plasma (pg/mL) | 2,094 (1,642 – 2,721) | 3,098 (2,512 – 4,370) | <0.001 |
| IL-6 in Plasma (pg/mL) | 348 (249 – 627) | 751 (502 – 1,317) | <0.001 |
| Angiopoietin-2 in Plasma (pg/mL) | 25,086 (21,942 – 28,835) | 17,937 (15,286 – 20,787) | <0.001 |
| ICAM-1 in Plasma (pg/mL) | 42,729 (35,758 – 58,015) | 38,978 (33,794 – 43,640) | 0.084 |
| MMP-8 in Plasma (pg/mL) | 486,903 (411,075 – 561,455) | 1,015,840 (758,550 – 1,335,338) | <0.001 |
| ***Data collected at 36h*** |  |  |  |
| SpO2 (%) | 85 (80 – 90) | 74 (59 – 83) | <0.001 |
| Body Weight (g) | 18.7 (17.4 – 19.5) | 19.2 (18.5 – 20.0) | 0.036 |
| Body Weight Change (%) | -11.6 (-13.6 – -9.1) | -4.2 (-7.5 – -2.5) | <0.001 |
| Body Temperature (°C) | 32.8 (29.7 – 34.0) | 25.0 (23.2 – 30.4) | <0.001 |
| Angiopoietin-2 in Plasma (pg/mL) | 31,495 (27,601 – 41,193) | 27,475 (19,494 – 33,405) | 0.014 |
| KC in Plasma (pg/mL) | 828 (613 – 2,910) | 8,280 (3,726 – 14,197) | <0.001 |
| sTNFR1 in Plasma (pg/mL) | 1,835 (1,342 – 2,521) | 2,170 (1,685 – 7,274) | 0.024 |
| IL-6 in Plasma (pg/mL) | 106 (41 – 410) | 3,261 (723 – 19,740) | <0.001 |
| ICAM-1 in Plasma (pg/mL) | 41,942 (30,550 – 51,634) | 41,206 (32,504 – 60,148) | 0.57 |
| MMP-8 in Plasma (pg/mL) | 397,585 (290,257 – 512,255) | 288,195 (199,197 – 446,668) | 0.060 |
| Bacterial Load in BAL (CFU/mL) | 2,300 (800 – 18,000) | 480,000 (60,000 – 1,500,000) | <0.001 |
| Protein Concentration in BAL (mg/mL) | 5.24 (4.20 – 6.73) | 9.16 (7.55 – 10.28) | <0.001 |
| KC in BAL (pg/mL) | 11 (11 – 5,454) | 13,888 (4,482 – 23,338) | <0.001 |
| IL-6 in BAL (pg/mL) | 4,302 (1,800 – 16,725) | 35,081 (18,324 – 45,900) | <0.001 |
| ***Mortality (%)*** | 1 (2) | 17 (35) | <0.001 |
| ^1^n (%); Median (Q1, Q3)  ^2^Pearson's Chi-squared test**;** Wilcoxon rank sum test. False discovery rate correction for multiple testing were applied. | | | |

**Abbreviations:** Angiopoietin-2: ANG-2; BAL: bronchoalveolar lavage; CFU: colony-forming unit; ICAM-1: intercellular adhesion molecule 1; IL-6: interleukin-6; KC: keratinocyte-derived chemokine; MMP-8: matrix metalloproteinase-8; Protein C: protein C; SpO₂: peripheral oxygen saturation; sTNFR1: soluble tumor necrosis factor receptor 1.

Table S11: Results of the Latent Class Analysis in all Mice (Development model and therapeutic model) (N=222).

| **Number of Class** | **BIC** | **Entropy** | **N1** | **N2** | **N3** | **N4** | **P-value** |
| --- | --- | --- | --- | --- | --- | --- | --- |
| 1 | 3,256 | - | 222 |  |  |  | - |
| 2 | 3,143 | 0.74 | 106 | 116 |  |  | <0.0001 |
| 3 | 3,141 | 0.8 | 109 | 12 | 101 |  | 0.1184 |
| 4 | 3,148 | 0.84 | 6 | 95 | 109 | 12 | 0.5121 |

**Abbreviations:** BIC: Bayesian information criteria

Table S12: Sensitivity analysis of the Latent Class Analysis in the Therapeutic Model of Pneumonia (N=222), adding Ang-2 and ICAM-1 as biomarker

| **Number of Class** | **BIC** | **Entropy** | **N1** | **N2** | **N3** | **N4** | **P-value** |
| --- | --- | --- | --- | --- | --- | --- | --- |
| 1 | 4,455.893 | - | 222 |  |  |  | - |
| 2 | 4,327.720 | 0.78 | 108 | 114 |  |  | <0.0001 |
| 3 | 4,327.505 | 0.8 | 90 | 105 | 27 |  | 0.3200 |
| 4 | 4,322.751 | 0.85 | 9 | 28 | 86 | 99 | 0.2029 |

**Abbreviations:** BIC: Bayesian information criteria

Table S13: Contingency table comparing LCA classification from the initial development phase model and the sensitivity model including Ang-2 and ICAM-1

|  | **Class 1 (Sensitivity model)** | **Class 2 (Sensitivity model)** |
| --- | --- | --- |
| **Class 1 (Primary model)** | 105 | 11 |
| **Class 2 (Primary model)** | 3 | 103 |

Table S14: Characteristics of groups before randomization in the Dexamethasone experiments

|  | **Overall** | | | **Less inflamed** | | **More inflamed** | |
| --- | --- | --- | --- | --- | --- | --- | --- |
| **Characteristic** | **Control  N = 28^1^** | **Dexamethasone  N = 27^1^** | **p-value^2^** | **Control  N = 13^1^** | **Dexamethasone  N = 15^1^** | **Control  N = 15^1^** | **Dexamethasone  N = 12^1^** |
| ***Data at baseline*** |  |  |  |  |  |  |  |
| Body Temperature (°C) | 37.6 (37.4 – 38.0) | 37.6 (37.2 – 37.9) | 0.66 | 37.5 (37.4 – 38.0) | 37.6 (37.2 – 38.0) | 37.6 (37.3 – 38.0) | 37.7 (37.4 – 37.9) |
| Body Weight (g) | 18.8 (17.7 – 20.1) | 18.8 (17.8 – 20.1) | 0.83 | 19.8 (18.8 – 20.5) | 20.0 (18.2 – 20.4) | 18.3 (17.0 – 19.8) | 18.1 (17.1 – 18.9) |
| ***Data at 18h (before randomization)*** |  |  |  |  |  |  |  |
| Body Temperature (°C) | 30.6 (28.9 – 34.3) | 31.5 (30.1 – 33.5) | 0.52 | 34.9 (33.1 – 35.2) | 33.4 (31.5 – 34.2) | 28.9 (28.0 – 30.0) | 30.4 (28.7 – 31.2) |
| Body Weight (g) | 18.8 (17.4 – 19.9) | 18.6 (17.4 – 20.7) | 0.92 | 18.9 (17.9 – 20.6) | 19.6 (17.7 – 20.9) | 17.7 (17.0 – 19.9) | 17.90(16.9 – 19.2) |
| SpO2 (%) | 93 (91 – 95) | 92 (90 – 94) | 0.11 | 93 (91 – 95) | 93 (90 – 95) | 93 (91 – 94) | 91 (90 – 93) |
| Bacteremia (%) | 16 (55) | 15 (56) | 0.98 | 2 (15) | 5 (33) | 13 (87) | 10 (83) |
| KC (pg/mL) | 6,530 (2,996 – 8,238) | 5,520 (3,081 – 7,744) | 0.47 | 5,440 (3,000 – 7,861) | 3,686 (2,844 – 6,681) | 6,902 (4,767 – 8,280) | 5,687 (4,135 – 8,385) |
| IL-6 (pg/mL) | 463 (303 – 942) | 440 (327 – 801) | 0.34 | 370 (256 – 463) | 362 (226 – 625) | 651 (463 – 1,762) | 529 (376 – 807) |
| TNFR1 (pg/mL) | 2,758 (1,800 – 3,914) | 2,210 (1,800 – 3,074) | 0.33 | 1,927 (1,422 – 2,758) | 2,083 (1,606 – 2,491) | 3,914 (2,603 – 5,150) | 3,318 (2,150 – 3,999) |
| MMP-8 (pg/mL) | 897,253 (622,815 – 1,272,867) | 783,665 (584,078 – 984,235) | 0.48 | 542,604 (473,479 – 649,296) | 593,788 (571,915 – 747,049) | 1,272,867 (1,107,351 – 2,012,931) | 1,035,471 (933,221 – 1,317,763) |
| Angiopoietin-2 (pg/mL) | 23,478 (18,555 – 29,919) | 23,688 (21,684 – 26,173) | 0.90 | 30,119 (25,762 – 34,357) | 24,625 (22,003 – 26,889) | 18,992 (17,119 – 20,937) | 22,108 (20,556 – 24,415) |
| ICAM-1 (pg/mL) | 40,852 (37,317 – 45,188) | 37,425 (31,781 – 40,907) | 0.037 | 40,524 (38,076 – 46,071) | 35,319 (29,655 – 39,816) | 41,398 (35,750 – 44,747) | 40,252 (36,076 – 44,802) |

**Abbreviations:** Angiopoietin-2: ANG-2; BAL: bronchoalveolar lavage; CFU: colony-forming unit; ICAM-1: intercellular adhesion molecule 1; IL-6: interleukin-6; KC: keratinocyte-derived chemokine; MMP-8: matrix metalloproteinase-8; Protein C: protein C; SpO₂: peripheral oxygen saturation; sTNFR1: soluble tumor necrosis factor receptor 1.

Table S15: Comparison of treatment effect (dexamethasone) at 36 hours stratified by class

|  | **Overall** | | | **Less inflamed** | | | **More inflamed** | | |
| --- | --- | --- | --- | --- | --- | --- | --- | --- | --- |
| **Characteristic** | **Control  N = 28** | **Dexamethasone  N = 27** | **p-value** | **Control  N = 13** | **Dexamethasone  N = 15** | **p-value** | **Control  N = 15** | **Dexamethasone  N = 12** | **p-value** |
| Body Weight (g) | 17.1 (16.2 – 18.7) | 16.8 (15.6 – 18.2) | 0.36 | 16.8 (16.0 – 18.5) | 17.0 (15.5 – 18.6) | 0.93 | 17.5 (16.3 – 19.2) | 16.5 (15.6 – 17.6) | 0.13 |
| Body Weight Change (%) | -11 (-13 – -6) | -11 (-14 – -8) | 0.43 | -13 (-16 – -12) | -13 (-15 – -10) | 0.63 | -4 (-6 – -3) | -7. (-9 – -5) | 0.053 |
| Body Temperature (°C) | 33.3 (32.1 – 34.6) | 33.6 (32.8 – 34.6) | 0.50 | 34.6 (33.5 – 34.7) | 34.0 (33.3 – 34.8) | 0.69 | 32.1 (26.7 – 33.1) | 31.5 (26.5 – 33.6) | 0.77 |
| SpO2 (%) | 86 (79 – 91) | 92 (89 – 94) | 0.004 | 89 (86 – 94) | 92 (89 – 96) | 0.29 | 78 (75 – 88) | 93 (89 – 94) | <0.001 |
| Protein Concentration in BAL (mg/mL) | 5.55 (3.98 – 7.24) | 4.06 (2.39 – 5.34) | 0.015 | 4.29 (3.57 – 5.07) | 3.06 (1.86 – 4.37) | 0.088 | 7.44 (5.57 – 9.62) | 5.22 (4.74 – 7.17) | 0.035 |
| Cell Count in BAL (10^6 cells/mL) | 1,505 (1,175 – 2,070) | 1,265 (795 – 2,045) | 0.28 | 1,600 (1,190 – 1,710) | 1,230 (946 – 1,390) | 0.20 | 1,485 (790 – 2,550) | 1,460 (524 – 2,280) | 0.78 |
| Neutrophils in Plasma (10^6 cells/mL) | 0.28 (0.19 – 0.48) | 0.33 (0.22 – 0.43) | 0.47 | 0.32 (0.22 – 0.61) | 0.32 (0.22 – 0.42) | 0.93 | 0.25 (0.18 – 0.39) | 0.33 (0.22 – 3.59) | 0.51 |
| Platelets in Plasma (10^6 cells/mL) | 609 (542 – 665) | 609 (552 – 661) | 0.93 | 643 (542 – 678) | 630 (576 – 665) | 0.64 | 577 (542 – 647) | 577 (524 – 604) | 0.97 |
| Bacterial Load in BAL (CFU/mL) | 2,500 (550 – 18,100) | 1,200 (200 – 15,500) | 0.68 | 600 (300 – 1,000) | 1,000 (200 – 7,200) | 0.69 | 7,500 (3,700 – 3,600,000) | 88,000 (4,000 – 200,000) | 0.77 |
| KC in Plasma (pg/mL) | 1,586 (551 – 5,898) | 833 (457 – 2,354) | 0.22 | 567 (463 – 1,096) | 623 (333 – 1,122) | 0.82 | 6,643 (2,528 – 31,430) | 4,385 (924 – 17,832) | 0.55 |
| TNFR1 in Plasma (pg/mL) | 2,065 (1,615 – 2,545) | 1,738 (1,334 – 2,043) | 0.065 | 2,127 (1,643 – 2,627) | 1,586 (1,231 – 2,045) | 0.12 | 2,065 (1,842 – 2,106) | 1,889 (1,462 – 2,041) | 0.18 |
| IL-6 in Plasma (pg/mL) | 317 (78 – 1,313) | 74 (26 – 432) | 0.043 | 90 (48 – 142) | 48 (26 – 86) | 0.069 | 1,370 (437 – 3,264) | 1,108 (114 – 2,008) | 0.44 |
| ICAM-1 in Plasma (pg/mL) | 43,566 (36,565 – 48,868) | 39,659 (30,237 – 47,289) | 0.22 | 37,217 (34,928 – 43,673) | 36,591 (30,974 – 44,747) | 0.44 | 45,396 (39,791 – 48,868) | 45,448 (29,501 – 50,732) | 0.74 |
| MMP-8 in Plasma (pg/mL) | 422,236 (339,168 – 643,346) | 1,136,120 (609,133 – 1,412,392) | <0.001 | 590,129 (396,930 – 823,757) | 1,271,236 (1,105,050 – 1,718,542) | <0.001 | 388,275 (253,050 – 433,729) | 472,706 (390,017 – 796,805) | 0.053 |
| Angiopoietin-2 in Plasma (pg/mL) | 36,993 (29,062 – 40,493) | 35,815 (27,593 – 49,543) | 0.81 | 34,632 (29,996 – 40,338) | 35,422 (29,186 – 49,093) | 0.78 | 38,866 (26,076 – 43,948) | 36,680 (25,332 – 52,230) | 0.78 |
| KC in BAL (pg/mL) | 1,839 (11 – 7,548) | 11 (11 – 2,040) | 0.049 | 11 (11 – 520) | 11 (11 – 11) | 0.20 | 8,388 (2,664 – 14,653) | 2,260 (1,703 – 6,797) | 0.094 |
| TNFR1 in BAL (pg/mL) | 2,452 (1,909 – 2,907) | 1,502 (1,236 – 2,014) | <0.001 | 2,123 (1,725 – 2,532) | 1,435 (1,214 – 1,550) | <0.001 | 2,763 (2,394 – 3,512) | 2,143 (1,785 – 2,767) | 0.053 |
| IL-6 in BAL (pg/mL) | 9,631 (2,290 – 23,890) | 2,778 (895 – 8,648) | 0.020 | 3,170 (1,019 – 5,197) | 1,248 (599 – 2,869) | 0.11 | 23,890 (13,093 – 32,498) | 9,545 (6,835 – 21,385) | 0.043 |
| Mortality | 5 (17) | 3 (11) | 0.71 | 0 (0%) | 0 (0%) | - | 5 (33) | 3 (25) | 0.70 |

**Abbreviations**: Angiopoietin-2: ANG-2; BAL: bronchoalveolar lavage; CFU: colony-forming unit; ICAM-1: intercellular adhesion molecule 1; IL-6: interleukin-6; KC: keratinocyte-derived chemokine; MMP-8: matrix metalloproteinase-8; Protein C: protein C; SpO₂: peripheral oxygen saturation; sTNFR1: soluble tumor necrosis factor receptor 1.

Table S16: Characteristics of groups before randomization in the IL-6 RA experiment

|  | **Overall** | | | **Less inflamed** | | **More inflamed** | |
| --- | --- | --- | --- | --- | --- | --- | --- |
| **Characteristic** | **Control  N = 30** | **IL-6 Receptor Antibody  N = 30** | **p-value** | **Control  N = 12** | **IL-6 Receptor Antibody  N = 14** | **Control  N = 18** | **IL-6 Receptor Antibody  N = 16** |
| ***At baseline (0h)*** |  |  |  |  |  |  |  |
| Body Temperature (°C) | 37.6 (37.1 – 37.9) | 37.5 (37.1 – 37.8) | 0.45 | 37.9 (37.1 – 38.0) | 37.4 (37.1 – 37.9) | 37.5 (37.1 – 37.7) | 37.5 (37.2 – 37.6) |
| Body Weight (g) | 19.2 (18.1 – 20.0) | 19.5 (18.7 – 20.2) | 0.25 | 18.8 (17.9 – 19.5) | 19.0 (18.2 – 20.0) | 19.67 (18.3 – 20.3) | 19.7 (19.1 – 20.6) |
| ***At 18h*** |  |  |  |  |  |  |  |
| Body Temperature (°C) | 31.9 (29.9 – 32.8) | 31.4 (30.1 – 32.8) | 0.77 | 33.8 (32.4 – 34.9) | 32.9 (32.6 – 33.7) | 30 (29.1 – 31.8) | 30.2 (28.7 – 31.0) |
| Body Weight (g) | 19.4 (18.7 – 20.0) | 19.4 (18.5 – 20.4) | 0.72 | 19.1 (18.3 – 19.5) | 19.2 (18.1 – 19.5) | 19.7 (18.9 – 20.4) | 20.1 (19.0 – 20.0) |
| SpO2 (%) | 92 (91 – 94) | 92 (90 – 95) | 0.95 | 93 (91 – 94) | 92 (90 – 93) | 92 (90 – 934) | 94 (90– 95) |
| Bacteremia (%) | 18 (60) | 20 (67) | 0.59 | 11 (92) | 12 (86) | 7 (39) | 8 (50) |
| KC (pg/mL) | 4,863 (3,773 – 6,472) | 4,230 (3,761 – 7,349) | 0.66 | 5,041 (4,532 – 6,464) | 4,090 (3,315 – 7,525) | 4,694 (3,689 – 7,397) | 4,484 (3,808 – 7,349) |
| IL-6 (pg/mL) | 512 (340 – 647) | 399 (316 – 598) | 0.24 | 494 (337 – 529) | 338 (261 – 538) | 574 (492 – 937) | 441 (355 – 750) |
| TNFR1 (pg/mL) | 2,293 (1,589 – 3,780) | 2,602 (1,968 – 3,227) | 0.36 | 1,476 (1,232 – 1,916) | 2,238 (1,958 – 2,856) | 3,543 (2,333 – 3,950) | 2,963 (2,303 – 4,247) |
| MMP-8 (pg/mL) | 924,984 (687,453 – 1,256,883) | 996,627 (653,897 – 1,217,925) | 0.74 | 643,805 (563,650 – 755,105) | 652,217 (566,200 – 929,997) | 1,155,234 (972,805 – 1,405,637) | 1,213,050 (1,094,075 – 1,586,394) |
| Angiopoietin-2 (pg/mL) | 21,116 (18,782 – 26,887) | 21,579 (18,546 – 24,480) | 0.89 | 26,962 (21,724 – 28,673) | 24,366 (21,887 – 27,484) | 19,173 (16,567 – 23,264) | 18,821 (16,647 – 21,579) |
| ICAM-1 (pg/mL) | 35,524 (33,732 – 38,982) | 34,910 (32,700 – 38,344) | 0.64 | 35,482 (31,982 – 38,904) | 34,926 (31,920 – 38,822) | 35,524 (34,046 – 38,982) | 34,910 (34,422 – 35,777) |

**Abbreviations**: Angiopoietin-2: ANG-2; BAL: bronchoalveolar lavage; CFU: colony-forming unit; ICAM-1: intercellular adhesion molecule 1; IL-6: interleukin-6; KC: keratinocyte-derived chemokine; MMP-8: matrix metalloproteinase-8; Protein C: protein C; SpO₂: peripheral oxygen saturation; sTNFR1: soluble tumor necrosis factor receptor 1.

Table S17: Comparison of treatment effect (IL-6 RA) at 36 hours stratified by class

|  | **Overall** | | | **Less inflamed** | | | | **More inflamed** | | |
| --- | --- | --- | --- | --- | --- | --- | --- | --- | --- | --- |
| **Characteristic** | **Control  N = 30** | **IL-6 Receptor Antibody  N = 30** | **p-value** | | **Control  N = 12** | **IL-6 Receptor Antibody  N = 14** | **p-value** | **Control  N = 18** | **IL-6 Receptor Antibody  N = 16** | **p-value** |
| ***Clinical data at 36h*** |  |  |  | |  |  |  |  |  |  |
| Body Weight (g) | 17.8 (16.8 – 18.8) | 17.8 (16.8 – 18.7) | 0.76 | | 17.0 (16.6 – 17.7) | 17.1 (16.2 – 17.4) | 0.90 | 18.3 (18.2 – 19.7) | 18.6 (18.0 – 19.0) | 0.60 |
| Body Weight Change (%) | -8 (-10 – -3) | -10 (-12 – -6) | 0.062 | | -9 (-11 – -8) | -10 (-13 – -9) | 0.23 | -3 (-8 – -1) | -6 (-10 – -4) | 0.088 |
| Body Temperature (°C) | 33.4 (28.6 – 34.2) | 33.4 (32.1 – 34.0) | 0.63 | | 33.8 (33.5 – 34.4) | 33.7 (32.8 – 34.5) | 0.84 | 32.3 (24.9 – 34.1) | 32.8 (31.0 – 33.8) | 0.45 |
| SpO2 (%) | 87 (81 – 89) | 89 (87 – 91) | 0.005 | | 87 (86 – 90) | 90 (89 – 92) | 0.16 | 81 (74 – 88) | 88 (87 – 89) | 0.014 |
| ***Plasma at 36h*** |  |  |  | |  |  |  |  |  |  |
| Neutrophils (10^6 cells/mL) | 0.18 (0.11 – 0.26) | 0.19 (0.11 – 0.26) | 0.94 | | 0.24 (0.14 – 0.43) | 0.24 (0.12 – 0.26) | 0.33 | 0.15 (0.10 – 0.20) | 0.16 (0.11 – 0.20) | 0.66 |
| Platelets (10^6 cells/mL) | 582 (541 – 617) | 578 (521 – 630) | 0.65 | | 601 (559 – 634) | 611 (540 – 686) | 0.60 | 558 (541 – 583) | 531 (505 – 578) | 0.22 |
| KC (pg/mL) | 2,299 (615 – 4,469) | 1,001 (549 – 2,813) | 0.18 | | 1,542 (562 – 2,620) | 624 (361 – 1,416) | 0.12 | 4,239 (962 – 18,205) | 1,283 (739 – 3,433) | 0.58 |
| TNFR1 (pg/mL) | 1,663 (1,391 – 2,053) | 2,490 (2,059 – 3,707) | <0.001 | | 1,751 (1,452 – 2,297) | 2,308 (2,059 – 2,822) | 0.023 | 1,586 (1,391 – 1,748) | 2,637 (2,182 – 3,707) | 0.001 |
| IL-6 (pg/mL) | 328 (74 – 681) | 1,395 (720 – 3,379) | <0.001 | | 198 (70 – 472) | 804 (368 – 2,123) | 0.002 | 586 (118 – 1,479) | 1,754 (1,395 – 4,302) | 0.029 |
| ICAM-1 (pg/mL) | 41,207 (34,247 – 51,172) | 44,742 (39,565 – 49,173) | 0.36 | | 43,616 (34,640 – 52,973) | 44,182 (38,142 – 47,871) | 0.86 | 40,041 (32,167 – 47,995) | 44,742 (41,537 – 52,651) | 0.14 |
| MMP-8 (pg/mL) | 446,937 (326,247 – 628,970) | 309,715 (265,075 – 483,885) | 0.013 | | 557,065 (377,688 – 762,576) | 349,745 (220,076 – 546,552) | 0.072 | 383,395 (294,436 – 549,117) | 303,352 (266,354 – 332,601) | 0.11 |
| Angiopoietin-2 (pg/mL) | 35,150 (28,458 – 43,799) | 37,504 (33,269 – 44,038) | 0.22 | | 36,051 (32,249 – 51,875) | 37,872 (33,269 – 39,949) | 0.98 | 33,521 (22,777 – 38,302) | 37,442 (34,149 – 44,870) | 0.14 |
| ***BAL at 36h*** |  |  |  | |  |  |  |  |  |  |
| Protein Concentration (mg/mL) | 6.61 (5.33 – 8.54) | 6.01 (3.18 – 6.87) | 0.040 | | 5.68 (4.04 – 6.68) | 3.36 (2.68 – 6.20) | 0.11 | 8.54 (6.10 – 9.78) | 6.11 (5.74 – 7.23) | 0.091 |
| Cell Count (10^6 cells/mL) | 1,580 (1,160 – 2,210) | 1,750 (1,350 – 2,580) | 0.25 | | 1,935 (1,330 – 2,445) | 1,620 (1,350 – 2,440) | 0.76 | 1,380 (882 – 1,980) | 2,260 (1,450 – 2,580) | 0.091 |
| Bacterial Load (CFU/mL) | 2,900 (600 – 11,000) | 1,400 (200 – 12,000) | 0.80 | | 1,550 (100 – 4,450) | 200 (100 – 4,000) | 0.75 | 3,100 (800 – 60,000) | 10,000 (1,400 – 30,000) | 0.84 |
| KC (pg/mL) | 3,484 (968 – 8,482) | 1,424 (11 – 3,907) | 0.11 | | 1,319 (11 – 3,167) | 11 (11 – 2,865) | 0.43 | 8,482 (3,484 – 16,186) | 2,321 (468 – 8,019) | 0.12 |
| TNFR1 (pg/mL) | 2,558 (2,322 – 2,827) | 1,787 (1,446 – 2,312) | 0.002 | | 2,427 (1,568 – 2,678) | 1,473 (1,121 – 1,960) | 0.027 | 2,634 (2,368 – 3,095) | 2,099 (1,787 – 2,396) | 0.022 |
| IL-6 (pg/mL) | 15,809 (3,607 – 36,279) | 12,863 (2,595 – 23,825) | 0.57 | | 7,659 (2,327 – 16,365) | 2,774 (2,397 – 16,971) | 0.78 | 36,279 (10,536 – 56,913) | 20,722 (12,863 – 29,609) | 0.65 |
| ICAM-1 (pg/mL) | 173,079 (135,414 – 203,876) | 138,369 (115,790 – 180,075) | 0.11 | | 155,172 (120,529 – 176,744) | 126,578 (108,624 – 171,415) | 0.25 | 175,742 (172,413 – 226,887) | 164,110 (130,168 – 207,583) | 0.20 |
| MMP-8 (pg/mL) | 31,098,925 (16,798,364 – 41,904,994) | 18,051,032 (9,164,857 – 22,979,576) | 0.013 | | 17,476,330 (12,076,503 – 27,819,183) | 9,853,027 (7,820,999 – 14,863,756) | 0.046 | 40,896,755 (32,614,108 – 54,365,236) | 22,884,251 (21,621,643 – 33,838,653) | 0.034 |
| Mortality | 5 (17) | 3 (10) | 0.71 | | 0 (0) | 0(0) | - | 5 (28) | 3 (19) | 0.69 |

**Abbreviations**: Angiopoietin-2: ANG-2; BAL: bronchoalveolar lavage; CFU: colony-forming unit; ICAM-1: intercellular adhesion molecule 1; IL-6: interleukin-6; KC: keratinocyte-derived chemokine; MMP-8: matrix metalloproteinase-8; Protein C: protein C; SpO₂: peripheral oxygen saturation; sTNFR1: soluble tumor necrosis factor receptor 1.

Table S 18: Interaction model for the dexamethasone experiment adjusted for baseline body weight

|  | SpO2 | | | Proteins in BAL | | |
| --- | --- | --- | --- | --- | --- | --- |
|  | **Beta** | **95% CI** | **p-value** | **Beta** | **95% CI** | **p-value** |
| Dexamethasone | 1.9 | -2.4 to 6.1 | 0.38 | -1.1 | -2.5 to 0.37 | 0.14 |
| More inflamed phenotype | -11 | -16 to -6.5 | <0.001 | 3.6 | 2.0 to 5.3 | <0.001 |
| Body Weight at 0h | 0.24 | -0.91 to 1.4 | 0.67 | -0.02 | -0.41 to 0.37 | 0.90 |
| Dexamethasone * More inflamed phenotype | 10 | 3.5 to 17 | 0.004 | -1.3 | -3.6 to 0.92 | 0.24 |
| Abbreviation: CI = Confidence Interval | | | |  |  |  |

Table S 19: Interaction model for the IL6-RA experiment adjusted for baseline body weight

|  | SpO2 | | | Proteins in BAL | | |
| --- | --- | --- | --- | --- | --- | --- |
|  | **Beta** | **95% CI** | **p-value** | **Beta** | **95% CI** | **p-value** |
| IL-6 Receptor Antibody | 1.8 | -2.7 to 6.4 | 0.42 | -1.3 | -2.8 to 0.11 | 0.070 |
| More inflamed phenotype | -7.9 | -13 to -3.1 | 0.002 | 2.3 | 0.78 to 3.8 | 0.004 |
| Body Weight at 0h | -0.60 | -2.1 to 0.87 | 0.42 | 0.42 | -0.05 to 0.89 | 0.080 |
| IL-6 Receptor Antibody * More inflamed phenotype | 6.4 | -0.01 to 13 | 0.050 | -0.36 | -2.4 to 1.7 | 0.72 |
| Abbreviation: CI = Confidence Interval | | | |  |  |  |

Table S 20: Mixed-effect model for the dexamethasone experiment incorporating batch as a random effect

|  | SpO2 | | BAL proteins | |
| --- | --- | --- | --- | --- |
|  | **Beta** | **95% CI** | **Beta** | **95% CI** |
| Dexamethasone | 1.8 | -2.3 to 5.9 | -1.1 | -2.4 to 0.31 |
| More inflamed phenotype | -12 | -16 to -7.1 | 3.6 | 2.1 to 5.2 |
| Dexamethasone * More inflamed | 10 | 3.7 to 17 | -1.3 | -3.5 to 0.83 |

Table S 21: Mixed-effect model for the IL6-RA experiment incorporating batch as a random effect

|  | SpO2 | | BAL proteins | |
| --- | --- | --- | --- | --- |
|  | **Beta** | **95% CI** | **Beta** | **95% CI** |
| IL-6 Receptor Antibody | 1.7 | -2.6 to 6.0 | -1.2 | -2.6 to 0.23 |
| More inflamed phenotype | -7.4 | -12 to -2.9 | 2.5 | 1.0 to 4.0 |
| IL-6 Receptor Antibody * More inflamed | 6.0 | -0.03 to 12 | -0.38 | -2.4 to 1.6 |
| Abbreviation: CI = Confidence Interval | | |  |  |

**s**
